# Bidirectional relationship between sexual arousal and (sex-related) disgust

**DOI:** 10.1371/journal.pone.0285596

**Published:** 2023-05-11

**Authors:** Guangju Wen, Caoyuan Niu, Yikang Zhang, Pekka Santtila

**Affiliations:** 1 School of Psychology and Cognitive Science, East China Normal University, Shanghai, People’s Republic of China; 2 Faculty of Arts and Sciences, NYU Shanghai, Shanghai, People’s Republic of China; 3 Faculty of Psychology and Neuroscience, Maastricht University, Maastricht, The Netherlands; Universidad Complutense Madrid, SPAIN

## Abstract

Sexual stimuli provoke both sexual arousal and disgust, and the coaction between these emotions determines sexually behavioral outcomes. The current research includes two experiments to explore the bidirectional relationship between sexual arousal toward erotic stimuli and disgust induced by sexual body fluids. Study 1 presented 234 participants (117 women) with sexual body (vs. neutral) fluids followed by erotic stimuli, and Study 2 presented 235 participants (117 women) with erotic (vs. neutral) videos followed by sexual body fluids (and a non-sex-related stimulus). Study 1 showed that exposure to sexual body fluids reduced sexual arousal and the likelihood of sexual engagement toward erotic stimuli in participants with high sexual disgust sensitivity but increased sexual arousal and the likelihood of sexual engagement in participants with low sexual disgust sensitivity, while Study 2 suggested that men exposed to erotic (vs. neutral) stimuli reported lower disgust, stronger sexual arousal state, and higher willingness to interact with the sexual body fluids. There was no relationship between subjective feelings of sexual arousal and disgust in these experiments, while the balance of sexual arousal and disgust toward sexual body fluids and erotic stimuli had a positive association. Also, exposure to erotic stimuli had no effect on reactions to generally disgusting stimulus, but feelings of sexual arousal toward erotic stimuli were positively associated with disgust induced by generally disgusting fluid. These findings suggest that Behavior Immune System regulates disgust to establish a balance between benefit and cost related to sex as well as provide insight into the process underlying sexual dysfunctions.

## Introduction

Sexual dysfunction is a common sexual complaint across different countries and cultures [1–3]. Evidence shows that 25–63% of women and 10–52% of men [4–8] experienced at least one of the sexual dysfunctions and suffered negative outcomes, including depression and anxiety [9]. From an evolutionary perspective, it has been argued that disgust may be involved in sexual dysfunctions given that body fluids such as saliva and semen exchanged during sexual activity involve a contagion risk [10]. Little empirical research has been conducted to explore this hypothesis so far. Thus, the present research explored the associations between disgust induced by sexual body fluids as well as a generally disgusting stimulus and sexual arousal.

### Disgust and sexuality

Disgust, one of the basic emotions, is part of the Behavioral Immune System and helps individuals avoid contamination and diseases by eliciting a strong urge to avoid or escape stimuli linked to them [11, 12]. However, sexual activity presents a challenge to the Behavioral Immune System, as it can be considered “disgusting” in at least two ways. First, sexual activity, involving the exchange of body fluids (e.g., saliva, sweat, semen, and vaginal secretions), increases the likelihood of being infected with a sexually transmitted infection [13, 14]. In fact, research shows that fluids, like saliva, are strong disgusting elicitors in non-sexual scenarios [15, 16]. Second, the apertures of the body involved in sexual activities, such as the mouth, vagina, and anus, are also the most sensitive to intrusion and contamination [15].

The information processing model of sexual arousal suggests that the appraisal of sexual stimuli affects the following sexual response [17]. People with negative attitudes toward sexuality experience negative emotions such as fear, shame, and disgust with the occurrence of sexual stimuli, and research has shown that individuals with sexual dysfunctions report higher disgust toward sexual stimuli [18] and pornography use is also related to negative feelings toward erotic stimuli [19]. Brain studies have also found that the presentation of sexual stimuli activates brain networks related to disgust stimuli [20].

Disgust associated with sex is, thus, a powerful emotion that seems to have the function of obstructing sexual activity. This raises the question of how people can engage in sex at all given that it elicits disgust. de Jong et al. (2013) developed a sexual arousal-disgust rival model to elucidate how sexual arousal and disgust induced by sexual stimuli affect the sexual responses of individuals. This model suggests that sexual stimuli elicit both sexual arousal and disgust. The feelings of sexual arousal motivate sexual approach behavior, but the elicited disgust instead facilitates avoidance of sexual stimuli [10]. To make the sexual activity happen successfully, individuals need to down-regulate the feelings of disgust induced by sexual stimuli. On the other hand, a sexually dysfunctional loop would occur in case disgust outweighs sexual arousal [10].

### Disgust inhibits sexual arousal

Research has found that disgust inhibits sexual arousal responses and shows a close relationship with sexual problems, especially in women [10]. Supporting this idea that disgust interferes with the development and persistence of sexual arousal [21], research has shown that women with vaginismus (vs. women without vaginismus) report greater overall disgust propensity measured by the Disgust Scale-Revised [22] and stronger disgust responses toward sexual stimuli [23, 24]. Women with female sexual interest/arousal disorder also showed higher heart rate deceleration, suggesting activation of a disgust-related defensive autonomic response, and reported higher feelings of disgust toward erotic stimuli than women without this disorder [18]. Similarly, men with (vs. without) erectile disorder also showed lower willingness to handle sexually contaminated stimuli [24].

Evidence from healthy populations also shows that disgust is associated with decreased sexual arousal. For instance, women reported lower sexual arousal toward the erotic film after being exposed to disgusting pictures [25], while men showed decreases in subjective and genital sexual arousal after smelling a disgusting odor [21]. These inhibiting effects also occurred unconsciously in men and women when a priming paradigm was adopted [26].

Although previous research provides evidence regarding the effects of disgust on sexual arousal, no studies have so far explored whether upregulation of specifically sex-related disgust, that is, disgust induced by sexual body fluids, would affect sexual arousal. Thus, the present study adopted sexual body fluids as sex-related disgusting stimuli, with the idea that these would adequately mimic the actual contamination-related stimuli associated with sexual activity, to explore if sex-related disgust would inhibit feelings of sexual arousal and sexual approach behavior toward subsequent erotic stimuli.

### Sexual arousal inhibits feelings of disgust

To engage in sexual activity, humans need to downregulate their feelings of disgust in order to make inherently disgusting sexual activities possible. Previous research has looked at the role of sexual arousal in inhibiting feelings of disgust toward sexual stimuli. Though several experiments have been conducted to explore the effects of sexual arousal on both sex-related disgust and non-sex-related disgust, the results remain somewhat contradictory.

Stevenson et al. (2011) found that male undergraduates reported lower feelings of disgust toward sex-related elicitors but not toward non-sex-related disgust cues after receiving a manipulation that upregulated their level of sexual arousal [27]. Women who received sexual arousal manipulation rated sex-related stimuli as less disgusting (decreases in disgust toward the non-sex-related stimuli were also found) and reported more willingness to finish both sex-related and non-sex-related disgusting tasks [16].

On the other hand, Pawlowska et al. (2020) used an emotion regulation strategy to explore if up-regulating sexual arousal or down-regulating disgust could affect feelings toward erotic videos, but the results showed that up-regulating sexual arousal had no effect on disgust and vice versa [28]. Similarly, evidence from individuals with obsessive-compulsive disorder related to contamination fear found no differences in behavioral or verbal responses to sex-related and non-related disgusting tasks after viewing sexually arousing films [29]. Lee et al. (2014) explored whether the upregulation of sexual arousal affected self-reported measures of trait disgust. Results showed that sexual arousal only reduced sexual disgust but increased pathogen disgust in women and had no effect on male responses [30]. Thus, the second aim of the present research was to explore if sexual arousal would inhibit both sex-related and non-sex-related disgust to explore if the suppressing effects of sexual arousal would be specific to sexual activity.

Understanding how the Behavior Immune System is designed to organize disgust to establish a balance between disgust and sexual arousal helps to understand the occurrence of sexual encounters and problems. The Behavior Immune System triggers disgust to induce avoidance behavior when individuals perceive pathogen cues, but it is also sensitive to the costs and benefits to adjust responses to these disgusting stimuli. For example, disgust temporarily decreases when sexual encounters occur in order for mating and reproduction. However, maladaptive disgust leads to sexual problems. Specifically, excessively high disgust reactions to sexual stimuli reduced individuals’ sexual desire, sexual arousal, and increased sexual avoidance [25, 31, 32]. On the other hand, excessively low disgust caused by sexual arousal might facilitate the development of unusual sexual interest (e.g., urophilia or coprophilia). Previous evidence has shown that men in the sexual arousal group (by imagery or self-stimulation) reported higher willingness to engage in uncommon sexual behaviors (e.g., “sex with extremely fat people”, “getting sexually excited from contact with animals”, “find the 12-year-old girl attractive”) [33]. Individuals’ willingness to engage in such behaviors and not experience feelings of disgust may have been inhibited by sexual arousal [27].

### Factors affecting the bidirectional relationship between sexual arousal and disgust

Previous results regarding the bidirectional relationship between sexual arousal and disgust are conflicting [16, 17]. One possibility is that individual differences moderate this bidirectional relationship. According to the sexual arousal and disgust rival model developed by de Jong et al. (2013), sexual stimuli elicit sexual arousal and disgust, while individuals’ trait characteristics may moderate the strength of the feelings elicited by sexual stimuli [10]. Specifically, disgust propensity may moderate the intensity of disgust generated by sexual stimuli, while sexual excitability may moderate the feelings of sexual arousal. Evidence has shown that people with high disgust sensitivity reported higher feelings of disgust toward disgusting stimuli, while those with high sexual inhibition and lower sexual excitability showed less sexual arousal toward erotic stimuli and may have a higher possibility of experiencing sexual difficulties [34, 35].

Gender differences, which show close relations with trait differences, also make contributions to individuals’ feelings toward stimuli and affect the interplay between sexual arousal and disgust. The parental investment theory suggests that women and men have a fundamental difference in obligatory costs related to reproduction [36]. Also, women have a higher risk of being infected by sexually transmitted diseases and of being assaulted sexually, resulting in them being more disgust sensitive and less excitable sexually [12, 37–39]. Similarly, previous research has found that, compared to men, women showed lower sexual excitation, higher sexual inhibition, and higher disgust sensitivity. Thus, these trait differences as well as gender differences may affect the effects of any manipulation of disgust and sexual arousal.

Interestingly, beyond the model from de Jong et al. (2013), research has also shown that disgust sensitivity, especially sexual disgust sensitivity, moderates the strength of sexual arousal elicited by sexual stimuli [40, 41]. This raises a question about whether sexual excitability moderates the disgust response toward disgusting stimuli as well as the effects of disgust on sexual arousal. Thus, the present research explored the effects of disgust sensitivity, sexual excitability, and gender to see if and how these factors affect the interplay between sexual arousal and disgust.

Another key feature of this model is that sexual stimuli induce both sexual arousal and disgust, and the balance between sexual arousal and disgust rather than the sole activation of sexual arousal or disgust determines individuals’ decision-making toward sexual stimuli [10]. Obviously, the use of experimental manipulation (e.g., being exposed to erotic stimuli) would include both of these emotions in the analysis, and the results might be counterintuitive when considering the side emotion elicited by the same stimuli (e.g., disgust elicited by erotic stimuli). Thus, the present research measured the subjective feelings of both sexual arousal and disgust toward each stimulus to explore how sexual arousal and disgust interplay with each other across stimuli.

Given that it is difficult to separate the activation of sexual arousal from disgust toward sex-related disgusting stimuli (and also the activation of disgust from sexual arousal toward erotic stimuli), and given the importance of the effects of the coactions between these two emotions on the behavioral outcomes toward erotic, sex-related, and non-sex-related stimuli, developing a variable that indicates the coaction between sexual arousal and disgust on behavior intention would be an alternative approach. The current study created a new variable named “sexual arousal state” that calculates the balance between sexual arousal and disgust toward the same stimuli to explore how this arousal state affected their reactions toward the subsequent stimuli.

### The current study

Previous research has found a bidirectional relationship between sexual arousal and disgust. However, the manipulations of previous experiments used to induce feelings of sex-related disgust (e.g., pictures of naked women with scars or behavioral tasks like lubricating a vibrator) might not adequately represent the actual stimuli present during sexual activity [16, 27]. To improve the ecological validity of the manipulations, sexual body fluids were adopted in the present research. In Study 1, participants first received a sex-related disgust (vs. neutral) manipulation and then rated erotic stimuli to explore if such disgust would adversely impact sexual arousal and sexual approach behavior. Study 2 presented an erotic (vs. neutral) video manipulation first and then sex-related disgust stimuli to explore if sexual arousal can suppress disgust to solve the question of how people can engage in “disgusting” sexual behavior. To explore if the effects of sexual arousal would be specific to sexual body fluids, we also included a non-sex-related disgusting stimulus as a control in Study 2.

The main hypotheses in Study 1 were:

H1.1: Exposing participants to sexual body (vs. neutral) fluid tasks would result in more disgust, higher sexual arousal, and weaker sexual arousal state toward the tasks, as well as lower willingness to finish the tasks. This disgust-inducing effect as well as the inhibiting effect on sexual arousal state and on willingness to finish the tasks would be more pronounced in women, whereas the sexual arousal-enhancing effect would be more pronounced in men.
H1.1b: Women would report more disgust, lower sexual arousal, weaker sexual arousal state, and lower willingness to finish the tasks compared to men.
H1.2: Sexual arousal, sexual arousal state, and the likelihood of sexual engagement elicited by subsequent erotic stimuli would be reduced, whereas disgust elicited by erotic stimuli would be increased after prior exposure to sexual body (vs. neutral) fluid tasks. These effects would be more pronounced in women than men.
H1.2b: Women would report more disgust, lower sexual arousal, weaker sexual arousal state, and lower likelihood of sexual engagement toward the erotic stimuli compared to men.
H1.3: The effects of sexual body fluid tasks on feelings toward subsequent erotic stimuli would be more pronounced in participants with less (vs. more) sexual excitability as well as those with more (vs. less) sexual disgust sensitivity.
H1.4: Feelings of disgust toward sexual body fluids would be negatively related to feelings of sexual arousal and the likelihood of sexual engagement toward erotic stimuli, while sexual arousal toward sexual body fluids would show positive association with these two variables.
H1.5: Sexual arousal state toward sexual body fluids would be positively related to sexual arousal state and the likelihood of sexual engagement toward the erotic stimuli, while sexual arousal state toward the erotic stimuli would mediate the association between the other two variables.

Our main hypotheses in Study 2 were:

H2.1: Exposing participants to erotic (vs. neutral) stimuli would result in higher feelings of sexual arousal, more disgust, and stronger sexual arousal state toward these stimuli. The inducing effects on sexual arousal and sexual arousal state would be more pronounced in men, whereas the disgust-inducing effect would be more pronounced in women.
H2.1b: Men would report higher sexual arousal, stronger sexual arousal state, and less disgust toward the erotic stimuli compared to women.
H2.2: Disgust elicited by subsequent sexual body fluid tasks would be reduced, whereas sexual arousal, sexual arousal state, and willingness to finish the tasks elicited would be increased after prior exposure to erotic (vs. neutral) stimuli. Also, disgust elicited by the generally disgusting fluid task would be reduced, whereas willingness to finish the generally disgusting fluid task would be increased after prior exposure to erotic (vs. neutral) stimuli. The effects of erotic stimuli on feelings toward subsequent sexual body fluid tasks and the generally disgusting task would be more pronounced in men compared to women.
H2.2b: Women would report more disgust, lower sexual arousal, weaker sexual arousal state, lower willingness toward the sexual body fluid tasks, as well as more disgust and lower willingness toward the generally disgusting task compared to men.
H2.3: The effects of erotic stimuli on feelings toward subsequent sexual body fluids tasks and the generally disgusting fluid task would be more pronounced in participants with more (vs. less) sexual excitability and those with less (vs. more) sexual disgust sensitivity.
H2.4: Sexual arousal toward erotic stimuli would be negatively related to disgust but positively related to the willingness to finish both sexual body fluids and generally disgusting fluid tasks, while disgust toward erotic stimuli would be positively related to feelings of disgust but negatively related to the willingness to finish both sexual body fluids and generally disgusting fluid tasks.
H2.5: Sexual arousal state toward erotic stimuli would be positively related to sexual arousal state toward sexual body fluid tasks and the willingness to finish these tasks, while sexual arousal state toward sexual body fluids tasks would mediate the relationship between these two emotions. Also, sexual arousal state toward erotic stimuli would be negatively related to disgust toward generally disgusting fluid task and positively related to the willingness to finish this task, while disgust toward the generally disgusting fluid task would mediate the relationship between sexual arousal state toward erotic stimuli and the willingness to finish the generally disgusting fluid task.

## Study 1. The effect of disgust induced by sexual body fluid tasks on reactions to erotic stimuli

### Materials and methods

#### Participants

The final sample in Study 1 included 234 participants (117 women) with a mean age of 23.19 (*SD* = 3.67). Demographic information regarding the participants is provided in Table 1.

**Table 1:** Demographic variables of the sample.

		Study 1 (*N* = 234)		Study 2 (*N* = 235)	
		Sexual body fluids group	Neutral fluids group	Erotic stimuli group	Neutral stimuli group
Age		23.08 (3.48)	23.29 (3.85)	22.32 (3.26)	22.25 (3.40)
Number of the sexual partners		0.95 (1.56)	0.96 (1.64)	1.05 (1.94)	1.21 (2.49)
Gender	Women	53	64	60	57
	Men	61	56	57	61
Education	≤ Senior high school	0	1	1	4
	> Senior high school	114	119	116	114
Occupation	Student	78	83	91	93
	Employed worker	34	31	23	23
	Other	2	6	3	2
Relationship length	Single	60	58	72	64
	< 1 month	2	2	1	4
	1-3 months	4	4	4	6
	4-6 months	8	7	4	5
	7-12 months	15	3	8	16
	1-2 years	18	23	8	12
	3-5 years	6	21	14	8
	6-10 years	1	2	5	3
	> 10 years	0	0	1	0
Income (¥)	No	42	58	56	60
	< 5k	43	37	44	42
	5k-8k	13	16	10	10
	9k-15k	12	8	5	5
	16k-30k	3	0	2	1
	31k-50k	1	1	0	0
	>50k	0	0	0	0
Health condition	Excellent	39	27	39	40
	Very good	48	56	48	51
	Good	19	24	20	20
	Fair	8	13	10	7
	Poor	0	0	0	0

Participants were recruited at East China Normal University and through social media (e.g., QQ, WeChat) and received 35CNY for their participation. Participants were included if they met the following criteria: 1) adult; 2) Chinese; 3) heterosexual; 4) not a psychology student or researcher; 5) not pregnant or breastfeeding; 6) no drinking (alcohol), coffee, or other drugs and no masturbation or other sexual activity for at least 12 hours before participating in the experiment.

#### Fluid type manipulation

*Sexual body fluid and neutral fluid tasks*. Eight 30-second videos depicting research assistants completing sex-related behavioral approach tasks were used to induce feelings of disgust related to sexual body fluids (also see Wen et al., 2023) [42]. Four kinds of sexual body fluids including sweat (sweat from the head and neck), saliva, body odor (sweat from an armpit), and genital secretions (semen for female participants and vaginal secretions for male participants) were used. These four ostensibly disgusting body fluids were all fake. Specifically, sweat and body odor was made of a mixture of water and an odorless, watery, and yellowish skin care product. Water sprayed from a plastic spray bottle was used for fake saliva. Semen-like lubricant (white) gel used frequently in adult videos was used to achieve the effect of fake semen, while colorless and transparent lubricant was used for vaginal secretions.

Four of the eight videos presenting a female research assistant completing the male-related body fluids were presented for female participants, while the other four videos presenting a male research assistant completing the female-related body fluids were presented for male participants. All these stimuli were put in a dish with a label indicating the type of fluid. Each video recorded the process where the assistant finished one of the behavioral tasks with three steps: 1) Observe the fluid in the dish, 2) Lift the dish and smell the fluid, and 3) Touch the fluid using their finger (while wearing a plastic glove).

In contrast, eight 30-second videos presenting the research assistant finishing the neutral behavioral approach tasks were used in the control group. The fluids used were sweet water, saline water, soda water, and water.

*Introductory videos for fluids*. Additionally, to increase the credibility of the fluid videos, we prepared two 30-second introductory videos to introduce information concerning how we obtained each of the fluids. The content of each of the two videos was the same with the only difference being the gender of the research assistant featured in each video. We presented the female version of the videos, recorded by a female research assistant, to the male participants, while we showed the male version of the videos, recorded by a male research assistant, to the female participants.

For the sweat introductory videos, the procedure involved wiping the assistants’ forehead, face, and neck with a cotton ball, which was then deposited into a dish for further use. The introductory videos for body odors, on the other hand, entailed placing a cotton ball under the assistants’ armpit for 15 seconds before removal (with a written notification to participants that the cotton ball had been under the armpit for 5 minutes), followed by depositing the cotton ball into a dish. Semen and vaginal secretions were collected sampling cups. The introductory video for the semen sample featured a research assistant who utilized a pipette to transfer and deposit a sample of “semen” from the sampling cup into a dish. For the introductory video of the vaginal secretions, a research assistant removed the secretions from a sampling cup using a cotton swab.

The introductory videos provided factual information about the preparation of the neutral fluids. Specifically, for water and soda water, the research assistants poured these liquids into a beaker and carefully used a dropper to add small amounts of the fluid into the dish. For saline water and sweet water, the assistants combined salt or sugar with water in a beaker, stirred the mixture with a glass rod, and then deposited a small amount of the solution into the dish using the dropper.

*Erotic stimuli*. Ten one-minute videos presenting static pictures of nude and semi-nude Asian models (four versions presenting pictures of male models for female participants and four presenting pictures of female models for male participants were created) were created to elicit sexual arousal in individuals. Each video included 15 pictures (7 ~ 8 nude pictures) and each picture was shown for 4 seconds. These pictures were selected from a Chinese erotic picture database randomly and have been shown to induce sexual arousal in the Chinese population [43]. Eight videos (four male versions and four female versions) were used in Experiment 1.

#### Psychometric instruments

*Socio-demographic data*. Socio-demographic data including gender, age, relationship status, sexual orientation, education, occupation, relationship length, monthly income, general physical health, and the total number of sexual partners was collected (see Table 1 for response options).

*The Three-Domain Disgust Scale (TDDS)*. The TDDS [44] including 21 items is designed to measure participants’ disgust sensitivity in three subscales: pathogen disgust (referring to avoidance of infectious microorganisms; e.g., “Seeing a cockroach run across the floor”), sexual disgust (referring to avoidance of sexual partners and behaviors that are detrimental to long-term reproductive success; e.g., “Finding out that someone you don’t like has sexual fantasies about you”), and moral disgust (referring to avoidance of social norm violators; e.g., “Deceiving a friend”). Participants rated the items on a 7- point Likert-type scale ranging from 0 (*not at all disgusting)* to 6 (*extremely disgusting*). The internal consistency reliability of the pathogen disgust, sexual disgust, and moral disgust subscales were good in both Study 1 (.67, .76, and .76 respectively) and Study 2 (.66, .81, and .74, respectively).

*The Disgust Propensity and Sensitivity Scale-Revised (DPSS-R)*. The DPSS-R [45] including 16 items measures people’s disgust propensity (the general tendency to respond with the emotion of disgust) via items such as “I avoid disgusting things” and disgust sensitivity (the impact of experiencing the emotion of disgust) via items such as “It scares me when I feel nauseous”, respectively on a 5-point Likert scale ranging from *never* (1) to *always* (5). In the current research, the disgust propensity subscale and sensitivity subscale had good internal consistencies in Study 1 (.73 and .76) and Study 2 (.78 and .79).

*The Disgust Scale-Revised (DS-R)*. The 25-item DS-R [22] measures individual differences in disgust sensitivity in terms of core (including food, animals, and body products), animal-reminder (including death and envelope violations), and contamination-based disgust (concerns about interpersonal transmission of essences). Participants circle true or false or rate how much they agree with statements pertaining to their tendencies regarding disgusting stimuli on a 3-point Likert scale (1 = *not*, 3 = *very*). The current experiment used total scores, with higher scores referring to higher disgust sensitivity. The Cronbach’s α of the whole scale in Study 1 and Study 2 were .80 and .77, respectively.

*The Sexual Desire Inventory (SDI)*. The 13-item SDI [46] includes two subscales measuring dyadic sexual desire (the expression of desire to behave sexually with another person; e.g., “During the last month, how often have you had sexual thoughts involving a partner?”) and solitary sexual desire (the interest in behaving sexually by oneself; e.g., “How strong is your desire to engage in sexual behavior by yourself?”). Participants were asked to answer questions regarding frequency on an 8-point Likert scale from 0 (*not at all*) to 7 (*more than once a day*) and rate other questions on a 9-point Likert scale from 0 (*not at all important*; *much less desire*; *no desire*) to 8 (*extremely important*; *much more desire*; *strong desire*). The Cronbach’s αs of the dyadic sexual desire, solitary sexual desire, and the total scale were good in Study 1 (.91, .87, and .94) and in Study 2 (.91, .86, and .93).

*The Sexual Inhibition/Sexual Excitation Scales – Short Form (SIS/SES-SF)*. SIS/SES-SF [47] consists of 14 items rated on a 4-point scale (1 = *strongly agree* to 4 = *strongly disagree*). Six items measure sexual excitation (SES; e.g., “When I think of a very attractive person, I easily become sexually aroused”), four items measure sexual inhibition scale 1 (SIS1; Inhibition because of the threat of sexual performance failure; e.g., “I cannot get aroused unless I focus exclusively on sexual stimulation”), and four items measure sexual inhibition scale 2 (SIS2; Inhibition because of the threat of sexual performance consequences; e.g., “If I realize there is a risk of catching a sexually transmitted disease, I am unlikely to stay sexually aroused”). Cronbach’s αs of SES, SIS1 and SIS2 were .82, .41, and .62 in Study 1, respectively, and .78, .50, and .62 in Study 2.

*The Positive and Negative Affect Schedule-Revised (PANAS-R)*. The PANAS-R [48] includes two 10-item scales measuring positive and negative effects on a 5-point scale of 1 (*not at all*) to 5 (*very much*). We selected 9 items randomly (including 4 positive items, 4 negative items, and 1 neutral item) and added two additional items (disgusting and sexually arousing). Only items measuring disgust and sexual arousal after viewing fluid task videos and erotic stimuli were used in the data analyses, while measurement of other items was used to disguise our experiment’s purpose.

*Willingness to finish the task*. A single item (“Please, imagine that you are the person in the video, to what extent would you be willing to finish this task”) rated on a 9-point scale (1 = *not at all*, 9 = *very*) was used to measure participants’ willingness to finish the task.

*Likelihood of sexual engagement*. A single item (“If an attractive opposite-sex person you met in the videos now wants to have sex with you, to what extent would you accept this invitation”) was rated on a graphic slider ranging from 0 (not at all) to 10 (very likely) was used to indicate their likelihood of sexual engagement.

*Sexual arousal state*. As mentioned before, we expected that the likelihood of sexual engagement would be affected by the coaction between sexual arousal and disgust. Thus, we developed a new variable named *sexual arousal state* describing the balance (difference) between sexual arousal and disgust toward the tasks (sexual arousal toward the fluid tasks minus disgust toward the fluid tasks) as well as toward erotic stimuli (sexual arousal toward erotic stimuli minus disgust toward erotic stimuli) measured via PANAS-R. A positive number indicated that sexual arousal was larger than disgust, while higher scores indicated a stronger arousal state.

#### Procedure

All the measurements were conducted online via *Qualtrics©* (www.qualtrics.com). Participants were assigned to the experimental group (videos related to sexual body fluids) or the control group (videos related to neutral fluids) randomly by *Qualtrics* and then viewed the corresponding videos. For each trial, participants were first asked to finish the PANAS-R measuring their baseline emotions. Then they were informed of the type of fluid in this round and viewed the corresponding introductory video (e.g., “In this round, you will watch a volunteer smell and touch the water; now watch the video below and learn how we got this fluid”) as well as the fluid task video (e.g., “Now please watch how the volunteer completes the task”). PANAS-R and one single item measuring their willingness to finish this task were then answered again. After this, they would view an erotic stimulus aimed at eliciting sexual arousal and again rate the PANAS-R and their likelihood of sexual engagement.

**Fig 1:**
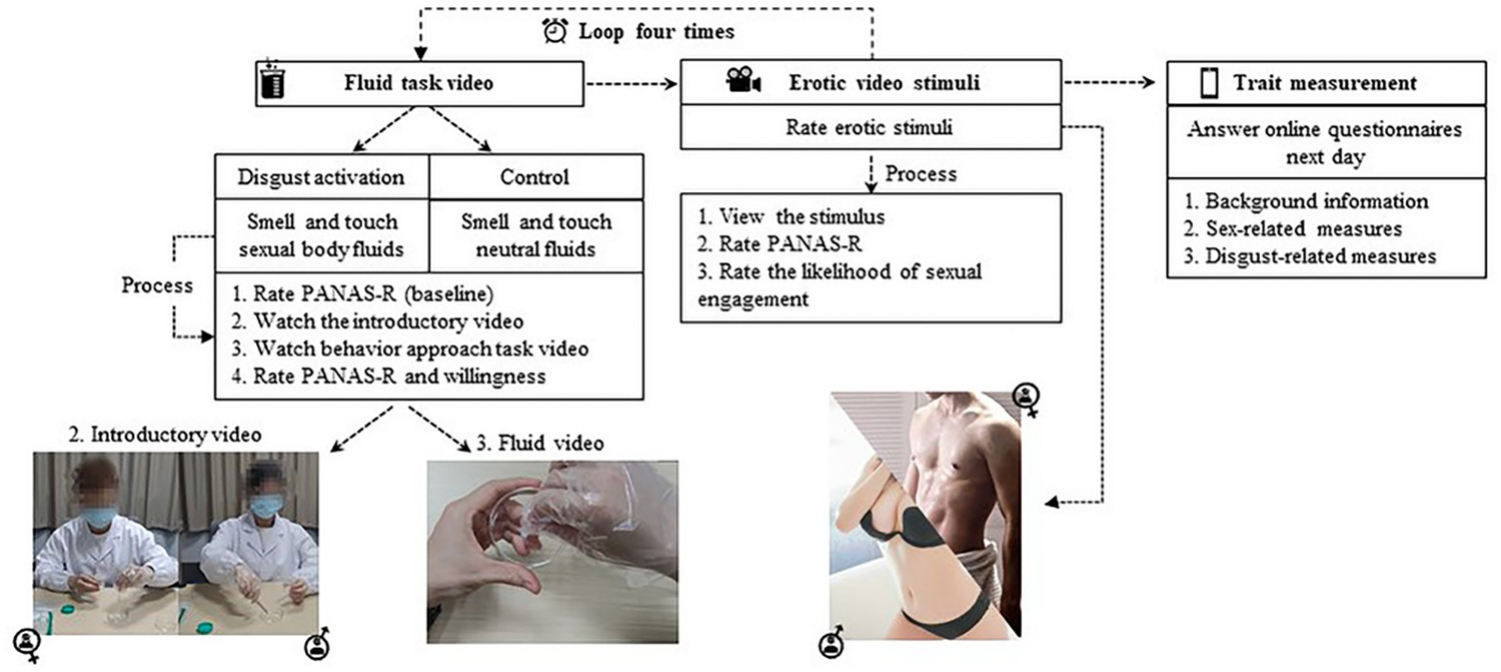
A flowchart presenting the procedure of Study 1.

Following this, participants needed to finish all four tasks in their group and then finish an online survey measuring their trait characteristics via *Wenjuanxing* (www.wjx.cn) the next day. A flowchart of the experiment is presented in Fig 1.

#### Data analysis

Mixed linear models using SPSS v25.0 were used to explore whether fluid type affected reactions to fluid videos and erotic stimuli. In these mixed linear models, the participants’ index was used as a Subjects variable and the different task types in each trial were used as Repeated variables. *P*-values (alpha level < .05) and 95% confidence intervals (not overlapping with zero) were used to indicate statistical significance.

First, a series of 2 (Fluid type: Sexual body fluid tasks vs. Neutral fluid tasks) x 2 (Gender: Women vs. Men) mixed linear models were conducted on reactions to fluid tasks to explore a) whether sexual body fluids tasks successfully induced feelings of disgust and lower willingness (and affected feelings of sexual arousal and sexual arousal state), b) whether these reactions showed significant gender differences, and c) whether the effects of fluid type would be different in men and women.

Second, a series of 2 (Fluid type: Sexual body fluid tasks vs. Neutral fluid tasks) x 2 (Gender: Women vs. Men) mixed linear models on reactions to erotic stimuli were conducted to explore a) whether sexual body fluids tasks would inhibit feelings of sexual arousal and the likelihood of sexual engagement (and feelings of disgust and sexual arousal state) toward erotic stimuli, b) whether these reactions showed significant gender differences, and c) whether the effects of fluid type would be different in men and women.

Third, a series of 2 (Fluid type: Sexual body fluid tasks vs. Neutral fluid tasks) x 2 (Traits: Low vs. High) x 2 (Gender: Women vs. Men) mixed linear models on reactions to erotic stimuli were conducted to explore a) whether the effects of fluid task type on reactions to erotic stimuli would be different in participants with low or high traits and b) whether the moderating effects of traits would be different in men and women. To limit the number of analyses, traits with the best reliability from disgust-related trait characteristics (sexual disgust, moral disgust, and pathogen disgust) and sexual-related trait characteristics (SES, SIS1, SIS2, sexual desire) were used in these analyses. In this case, sexual disgust and SES were included. The means of the traits were used as threshold values for high and low grouping. Participants scoring above the means on traits were allocated to the high group, whereas others were allocated to the low group.

Next, subjective ratings of sexual arousal and disgust of individuals were used to examine if there was a negatively bidirectional relationship between these two emotions. Data from the experimental group was used because no disgust or sexual arousal was expected to be experienced in the control group. Feelings and reactions toward the same question from four different trials were merged into one latent variable and SmartPLS 3.0 was used to do the regression and mediation analyses.

Fourth, we examined if disgust toward sexual body fluids was negatively related to sexual arousal and the likelihood of sexual engagement toward erotic stimuli. We were also interested in if sexual arousal toward sexual body fluids was positively related to sexual arousal and the likelihood of sexual engagement toward erotic stimuli.

Fifth, to explore the coaction between sexual arousal and disgust toward sexual body fluids on reactions to erotic stimuli, we explored if a) sexual arousal state toward sexual body fluids was positively related to sexual arousal state and the likelihood of sexual engagement toward erotic stimuli and b) if sexual arousal state toward erotic stimuli mediated the relationship between sexual arousal state toward sexual body fluids and the likelihood of sexual engagement toward erotic stimuli.

#### Ethical statement and pre-registration

The current study involving human participants received approval from the Institutional Review Board of NYU Shanghai (Approval Number is 2021-020-NYUSH-Zhongbei). Written informed consent for participation was waived for this study in accordance with national legislation and institutional requirements. Before completing the experiment, participants were required to read and acknowledge an electronic consent form that displayed all details of the experiments (e.g., eligibility criteria, experiment procedures, the contact information of the Institutional Review Board), and indicate their decision to participate in the experiment by clicking on the "Yes, I agree to participate (and confirm that I am eligible for the study)" button to proceed with the survey. Before the data collection began, a pre-registration was submitted to ASPREDICTED.COM (https://aspredicted.org/WG2_3PD).

### Results

#### Do sexual body fluids induce feelings of disgust and lower willingness?

Table 2 presents the effects of fluid type and gender on reactions to fluid tasks and erotic stimuli from the mixed linear models.

**Table 2:** The effect of fluid type and gender on reactions to fluid videos and erotic stimuli.

**Outcome (Reactions to fluid videos)**	**Predictor**	***df***	***F***	***p***
Sexual arousal	Fluid type	864.48	1.99	.159
Disgust		903.06	251.01	< .001
Willingness to finish the tasks		926.51	593.51	< .001
Sexual arousal state		874.17	164.72	< .001
Sexual arousal	Gender	864.48	31.39	< .001
Disgust		903.06	5.57	.018
Willingness to finish the tasks		926.51	2.00	.157
Sexual arousal state		874.17	22.99	< .001
Sexual arousal	Fluid type*Gender	864.48	7.57	.006
Disgust		903.06	6.25	.013
Willingness to finish the tasks		926.51	4.67	.031
Sexual arousal state		874.17	10.75	.001
**Outcome (Reactions to erotic stimuli)**	**Predictor**	***df***	***F***	***p***
Sexual arousal	Fluid type	925.40	0.52	.470
Disgust		910.86	0.27	.602
Likelihood of sexual engagement		872.87	0.87	.351
Sexual arousal state		921.42	0.06	.801
Sexual arousal	Gender	925.40	80.89	< .001
Disgust		910.86	129.71	< .001
Likelihood of sexual engagement		872.87	146.45	< .001
Sexual arousal state		921.42	177.44	< .001
Sexual arousal	Fluid type*Gender	925.40	0.95	.330
Disgust		910.86	0.99	.320
Likelihood of sexual engagement		872.87	0.20	.656
Sexual arousal state		921.42	0.03	.875

*Note*: Fluid type refers to either sexual body fluids or neutral fluids that participants were exposed to prior to being shown erotic stimuli in subsequent tasks.

**H1.1: Exposing participants to sexual body (vs. neutral) fluid tasks would result in more disgust, higher sexual arousal, weaker sexual arousal state toward the tasks, and lower willingness to finish the tasks. This disgust-inducing effect and inhibiting effects on sexual arousal state, willingness to finish the tasks would be more pronounced in women, whereas the sexual arousal-enhancing effect would be more pronounced in men**.

There was an effect of fluid type on feelings of disgust, sexual arousal state toward the tasks, and the willingness to finish them (*ps* < .001). Participants viewing sexual body (vs. neutral) fluid tasks reported more disgust (*M*_BATBodyfluid_ = 2.17, *SD*_BATBodyfluid_ = 1.21; *M*_BATNeutral_ = 1.19, *SD*_BATNeutral_ = 0.50), weaker sexual arousal state toward the tasks (*M*_BATBodyfluid_ = -0.92, *SD*_BATBodyfluid_ = 1.39; *M*_BATNeutral_ = -0.04, *SD*_BATNeutral_ = 0.60), and a lower willingness to finish them (*M*_BATBodyfluid_ = 3.82, *SD*_BATBodyfluid_ = 2.41, *M*_BATNeutral_ = 7.36, *SD*_BATNeutral_ = 2.11). However, there was no effect of fluid type on feelings of sexual arousal toward the tasks (*M*_BATBodyfluid_ = 1.25, *SD*_BATBodyfluid_ = 0.60; *M*_BATNeutral_ = 1.15, *SD*_BATNeutral_ = 0.47; *p* = .159) even though the means were in the expected direction.

There was a significant interaction between gender and fluid type on sexual arousal toward the tasks (*p* = .006). Men viewing sexual body (vs. neutral) fluid tasks reported higher sexual arousal (*M*_BATBodyfluid_ = 1.39, *SD*_BATBodyfluid_ = 0.71; *M*_BATNeutral_ = 1.21, *SD*_BATNeutral_ = 0.57; *p* = .003), while there was no significant fluid type effect in women (*M*_BATBodyfluid_ = 1.08, *SD*_BATBodyfluid_ = 0.37; *M*_BATNeutral_ = 1.11, *SD*_BATNeutral_ = 0.37; *p* = .344). There were significant interactions between gender and fluid type on feelings of disgust (*p* = .013), sexual arousal state (*p* = .001), and willingness to finish the tasks (*p* = .031). Post hoc analyses showed that both men and women who viewed sexual body (vs. neutral) fluid tasks reported more disgust, weaker sexual arousal state toward the tasks, and a lower willingness to finish the tasks (*ps* < .001). Instead, men viewing the sexual body fluid (vs. women viewing the sexual body fluid) reported less disgust (*M*_Men_ = 2.03, *SD*_Men_ = 1.12; *M*_Women_ = 2.33, *SD*_Women_ = 1.29; *p* = .001), higher sexual arousal state toward the tasks (*M*_Men_ = -.64, *SD*_Men_ = 1.38; *M*_Women_ = -1.24, *SD*_Women_ = 1.33; *p* < .001) and higher willingness to finish the tasks (*M*_Men_ = 4.07, *SD*_Men_ = 2.38; *M*_Women_ = 3.53, *SD*_Women_ = 2.41; *p* = .013), while there was no significant gender effect on feelings of disgust, sexual arousal state toward the tasks and willingness to finish the tasks (*ps* > .278) in participants viewing the neutral fluid.

**H1.1b: Women would report more disgust, lower sexual arousal, weaker sexual arousal state, and lower willingness to finish the tasks compared to men**.

Gender had a main effect on feelings of disgust (*p* = .018), sexual arousal (*p* < .001), and sexual arousal state toward the tasks (*p* < .001). Women reported more disgust (*M*_Men_ = 1.63, *SD*_Men_ = 0.96; *M*_Women_ = 1.70, *SD*_Women_ = 1.11), weaker sexual arousal state toward the tasks (*M*_Men_ =-0.33, *SD*_Men_ = 1.15; *M*_Women_ = -0.60, *SD*_Women_ = 1.12), and lower sexual arousal toward the tasks (*M*_Men_ = 1.30, *SD*_Men_ = 0.66; *M*_Women_ = 1.10, *SD*_Women_ = 0.37). There was no gender difference in the willingness to finish the tasks (*p* = .157).

#### Do sexual body fluids affect reactions toward erotic stimuli?

**H1.2: Sexual arousal, sexual arousal state, and the likelihood of sexual engagement elicited by subsequent erotic stimuli would be reduced, whereas disgust elicited by erotic stimuli would be increased after prior exposure to sexual body fluid (vs. neutral) tasks. The effects of sexual body fluid tasks on feelings toward subsequent erotic stimuli would be more pronounced in women than men**.

As presented in Table 2, fluid type did not affect sexual arousal, disgust, sexual arousal state, or the likelihood of sexual engagement toward erotic stimuli (*ps* > .351).

There was no significant interaction between gender and fluid type on sexual arousal, disgust, sexual arousal state, and the likelihood of sexual engagement with the erotic stimuli (*ps* > .320).

**H1.2b: Women would report more disgust, lower sexual arousal, weaker sexual arousal state, and lower likelihood of sexual engagement toward the erotic stimuli compared to men**.

Gender had main effects on feelings of sexual arousal, disgust, sexual arousal state, and the likelihood of sexual engagement (*ps* < .001). Men reported higher sexual arousal (*M*_Men_ = 2.63, *SD*_Men_ = 1.19; *M*_Women_ = 1.99, *SD*_Women_ = 1.00), less disgust (*M*_Men_ = 1.28, *SD*_Men_ = 0.58; *M*_Women_ = 1.92, *SD*_Women_ = 1.07), stronger sexual arousal state (*M*_Men_ = 1.35, *SD*_Men_ = 1.32; *M*_Women_ = 0.06, *SD*_Women_ = 1.61), and higher likelihood of sexual engagement with the erotic stimuli (*M*_Men_ = 5.66, *SD*_Men_ = 3.10; *M*_Women_ = 3.26, *SD*_Women_ = 2.70).

#### Do sexuality and disgust-related trait characteristics moderate the effects of disgust induced by sexual body fluids on reactions to erotic stimuli?

Table 3 presents the results of the interactions between fluid type, trait characteristics, and gender on reactions to erotic stimuli.

**Table 3:** The interaction between fluid type, trait characteristics, and gender on reactions to erotic stimuli.

Outcome^a^	Predictor	*df*	*F*	*p*
Sexual arousal	Fluid type*Sexual excitability	779.36	< .001	.997
Disgust		766.41	0.22	.637
Likelihood of sexual engagement		736.13	2.66	.103
Sexual arousal state		777.21	0.08	.780
Sexual arousal	Fluid type*Sexual excitability*Gender	779.36	0.54	.463
Disgust		766.41	0.47	.494
Likelihood of sexual engagement		736.13	0.58	.447
Sexual arousal state		777.21	< .001	.956
Sexual arousal	Fluid type*Sexual disgust sensitivity	780.34	15.93	< .001
Disgust		763.22	3.30	.070
Likelihood of sexual engagement		763.22	3.30	.009
Sexual arousal state		777.85	3.80	.052
Sexual arousal	Fluid type*Sexual disgust sensitivity*Gender	780.34	0.97	.324
Disgust		763.22	1.77	.184
Likelihood of sexual engagement		763.22	1.77	.017
Sexual arousal state		777.85	1.97	.161

Note

^a^ = Reactions to the erotic stimuli. Fluid type refers to either sexual body fluids or neutral fluids that participants were exposed to prior to being shown erotic stimuli in subsequent tasks.

**H1.3 The effects of sexual body fluid tasks on feelings toward subsequent erotic stimuli would be more pronounced in participants with less (vs. more) sexual excitability as well as those with more (vs. less) sexual disgust sensitivity**.

Regarding the moderating effect of sexual excitability, there was no interaction between fluid type and sexual excitability (*ps* > .103) or interaction between fluid type, sexual excitability, and gender (*ps* > .463) on sexual arousal, disgust, sexual arousal state, or the likelihood of sexual engagement with the erotic stimuli.

**Fig 2:**
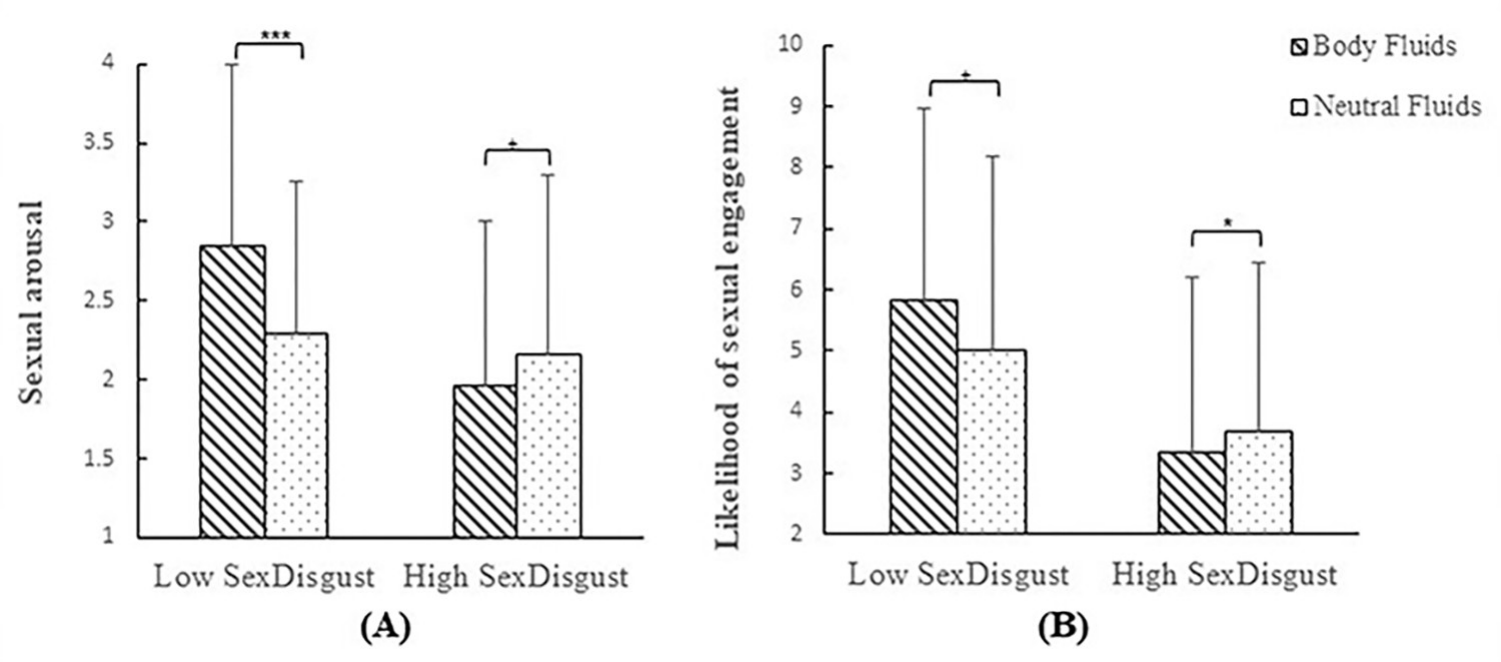
The interaction between fluid type and sexual disgust sensitivity on reactions to erotic stimuli. *Note*: + *p* < .10, * *p* < .05, *** *p* < .001.

Regarding the moderating effect of sexual disgust sensitivity, there was a significant interaction between fluid type and sexual disgust sensitivity on sexual arousal (*p* < .001; See Fig 2A) and the likelihood of sexual engagement (*p* = .009; See Fig 2B). Participants with low sexual disgust sensitivity viewing sexual body fluid (vs. neutral) tasks reported higher sexual arousal (*M*_BATBodyfluid_ =2.85, *SD*_BATBodyfluid_ =1.32; *M*_BATNeutral_ = 2.29, *SD*_BATNeutral_ = 0.97; *p* < .001) and higher likelihood of sexual engagement (*M*_BATBodyfluid_ = 5.83, *SD*_BATBodyfluid_ = 3.15; *M*_BATNeutral_ = 5.02, *SD*_BATNeutral_ = 3.16; *p* =.094), while participants with high sexual disgust sensitivity viewing sexual body fluid (vs. neutral) tasks reported lower sexual arousal (*M*_BATBodyfluid_ = 1.96, *SD*_BATBodyfluid_ =1.05; *M*_BATNeutral_ = 2.16, *SD*_BATNeutral_ = 1.14; *p* = .081) and lower likelihood of sexual engagement (*M*_BATBodyfluid_ = 3.33, *SD*_BATBodyfluid_ = 2.89; *M*_BATNeutral_ = 3.69, *SD*_BATNeutral_ = 2.77; *p* = .043). There was no interaction between fluid type and sexual disgust sensitivity on disgust or sexual arousal state toward erotic stimuli (*ps* > .052).

There was also a significant interaction between task type, sexual disgust sensitivity, and gender (*p* = .017) on the likelihood of sexual engagement. Men with low sexual disgust sensitivity viewing sexual body fluid (vs. neutral) tasks reported higher likelihood of sexual engagement (*M*_BATBodyfluid_ = 6.46, *SD*_BATBodyfluid_ = 3.05; *M*_BATNeutral_ = 5.63, *SD*_BATNeutral_ = 3.08; *p* = .018), while men with high sexual disgust sensitivity viewing sexual body fluid (vs. neutral) tasks reported lower likelihood of sexual engagement (*M*_BATBodyfluid_ = 3.66, *SD*_BATBodyfluid_ = 3.38; *M*_BATNeutral_ = 5.20, *SD*_BATNeutral_ = 2.52; *p* = .007). There was no fluid type difference in women with low (*p* = .599) or high (*p* = .554) sexual disgust sensitivity.

#### Are the subjective feelings of disgust and sexual arousal induced by sexual body fluid tasks associated with sexual arousal and the likelihood of sexual engagement toward erotic stimuli?

**Fig 3:**
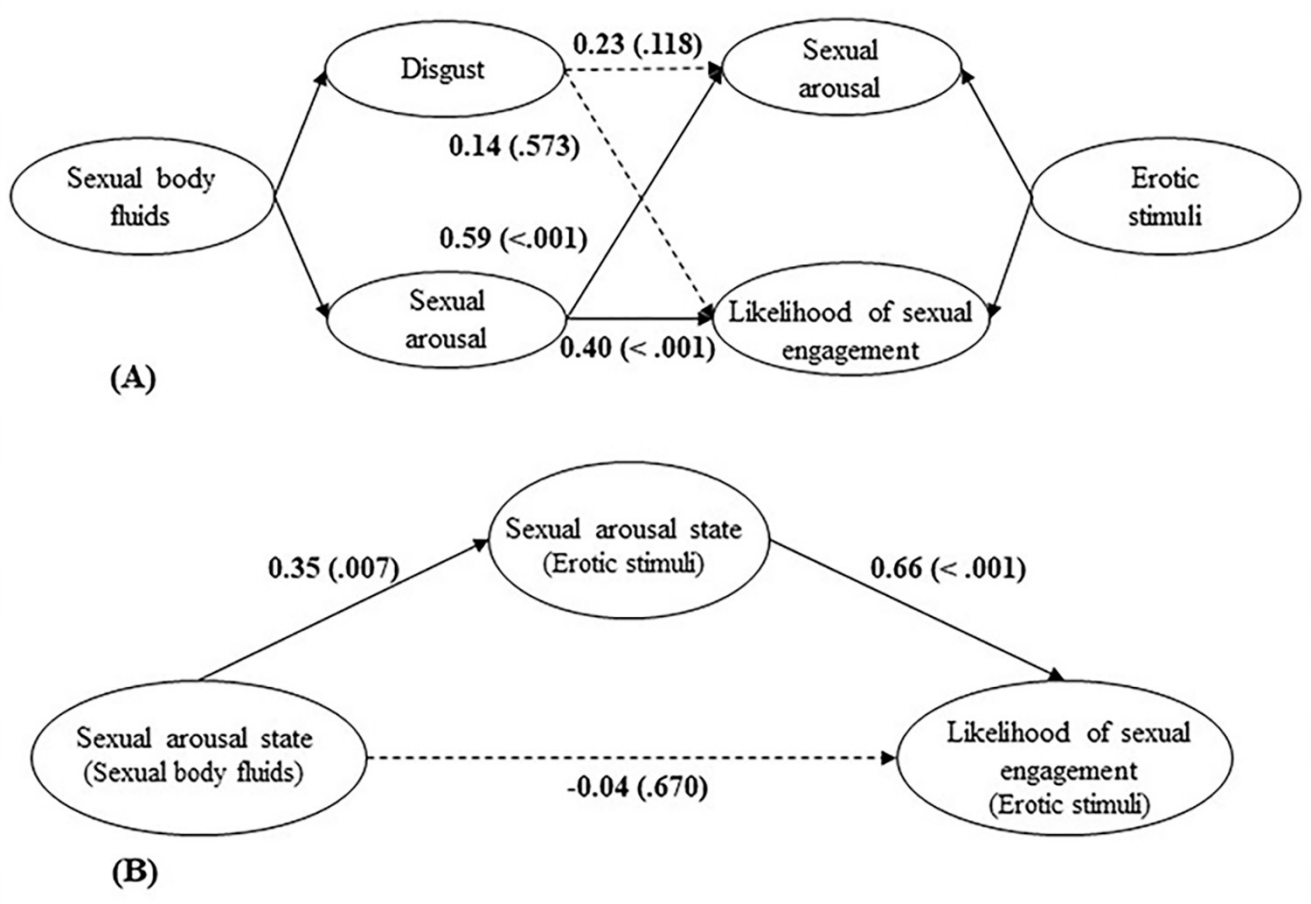
The associations between subjective feelings toward sexual body fluids and reactions to erotic stimuli. *Note*: Number outside the brackets = *β*; Number in the brackets = *p*-value.

Fig 3 presents the associations between subjective feelings toward sexual body fluids and reactions to erotic stimuli.

**H 1.4.1 Feelings of disgust toward sexual body fluids would be negatively related to sexual arousal and the likelihood of sexual engagement toward erotic stimuli**.

As presented in Fig 3A, feelings of disgust toward sexual body fluids had no relationship with sexual arousal and the likelihood of sexual engagement toward erotic stimuli (*ps* > .118).

**H 1.4.2 Feelings of sexual arousal toward sexual body fluids would be positively related to sexual arousal and the likelihood of sexual engagement toward erotic stimuli**.

Results showed that feelings of sexual arousal toward sexual body fluids were positively related to sexual arousal and the likelihood of sexual engagement toward erotic stimuli (*ps* < .001)

#### Does sexual arousal state toward sexual body fluids affect sexual arousal state and the likelihood of sexual engagement toward erotic stimuli?

H 1.5.1 The sexual arousal state toward sexual body fluids would be positively related to sexual arousal state and the likelihood of sexual engagement toward erotic stimuli.

Regression analyses showed that sexual arousal state toward sexual body fluids was positively related to sexual arousal state toward erotic stimuli (*β* = 0.41, *p* < .001) but not related to the likelihood of sexual engagement (*β* = 0.18, *p* = .146)

**H 1.5.2 Sexual arousal state toward sexual body fluids would affect the likelihood of sexual engagement toward erotic stimuli via its effect on sexual arousal state toward erotic stimuli**.

As presented in Fig 3B, sexual arousal state toward sexual body fluids was positively related to sexual arousal state toward erotic stimuli (*p* = .007), and sexual arousal state toward erotic stimuli was positively related to the likelihood of sexual engagement toward erotic stimuli (*p* < .001). Sexual arousal state toward erotic stimuli mediated the relationship between sexual arousal state toward sexual body fluids and the likelihood of sexual engagement and the standardized indirect effect was 0.23 (95% CI [.18, 1.45]).

## Study 2. The effect of sexual arousal induced by erotic stimuli on reactions to sexually disgusting body fluids and to a generally disgusting material

### Materials and methods

#### Participants

The final sample in Study 2 included 235 participants (117 women) using the same method and inclusion criteria as Experiment 1 with a mean age of 22.28 (*SD* = 3.32). These participants’ demographic data are also provided in Table 1.

#### Erotic stimuli and neutral stimuli

Ten one-minute videos presenting static pictures of nude and semi-nude Asian models (five presenting pictures of male models for female participants and five presenting pictures of female models for male participants) created in Study 1 were used to elicit sexual arousal of individuals in the experimental group.

In contrast, five one-minute videos presenting static pictures of landscapes were used for male and female participants in the neutral group. Each video included 15 pictures and each picture lasts for 4 seconds.

#### Fluid tasks video

*Sexual body fluid tasks and generally disgusting fluid task*. The same eight 30-second videos regarding four sexual body fluids used in Study 1 were used to induce sex-related disgust. Four videos presenting a female research assistant completing the male-related body fluids were presented for female participants while four videos presenting a male research assistant completing the female-related body fluids were presented for male participants.

Also, two 30-second videos presenting the male and female research assistants finishing the generally disgusting fluid task were used to induce non-sex-related disgust. The stimulus in the video was the remnants of a meal. The process of this task was the same as the sexual body fluid tasks, recording the research assistant smelling and touching this material by wearing a glove.

*Introductory videos for fluids*. Besides the introductory videos for the sexual body fluids, we also prepared two new 30-second (a male version and a female version) introductory videos to introduce how we obtained the generally disgusting material. The videos recorded the process where the assistants scooped the remnant of a meal from a beaker into a dish.

#### Measures

*Psychometric instruments*. Study 2 used the same psychometric instrument as in Study 1. However, the participants did not report their likelihood of sexual engagement after viewing the erotic or neutral video stimuli.

*Procedures*. All the measurements were conducted online via *Qualtrics*. Participants were assigned to the experimental group (erotic stimuli) or the control group (neutral stimuli) randomly by *Qualtrics* and then viewed the corresponding videos. For each trial, participants were asked to finish the PANAS-R measuring their baseline emotion first, view an erotic or neutral video stimulus to receive our manipulation, and then rated the PANAS-R again. After this, participants needed to view the fluid task videos. They first were informed of the type of fluid in this round and viewed the corresponding introductory video (e.g., “In this round, you will watch a volunteer smell and touch the sweat; now watch the video below and learn how we got this fluid”) as well as the behavioral approach task (e.g., “Now please watch how the volunteer to complete the task”). PANAS-R and one single item measuring their willingness to finish this task were answered again.

**Fig 4:**
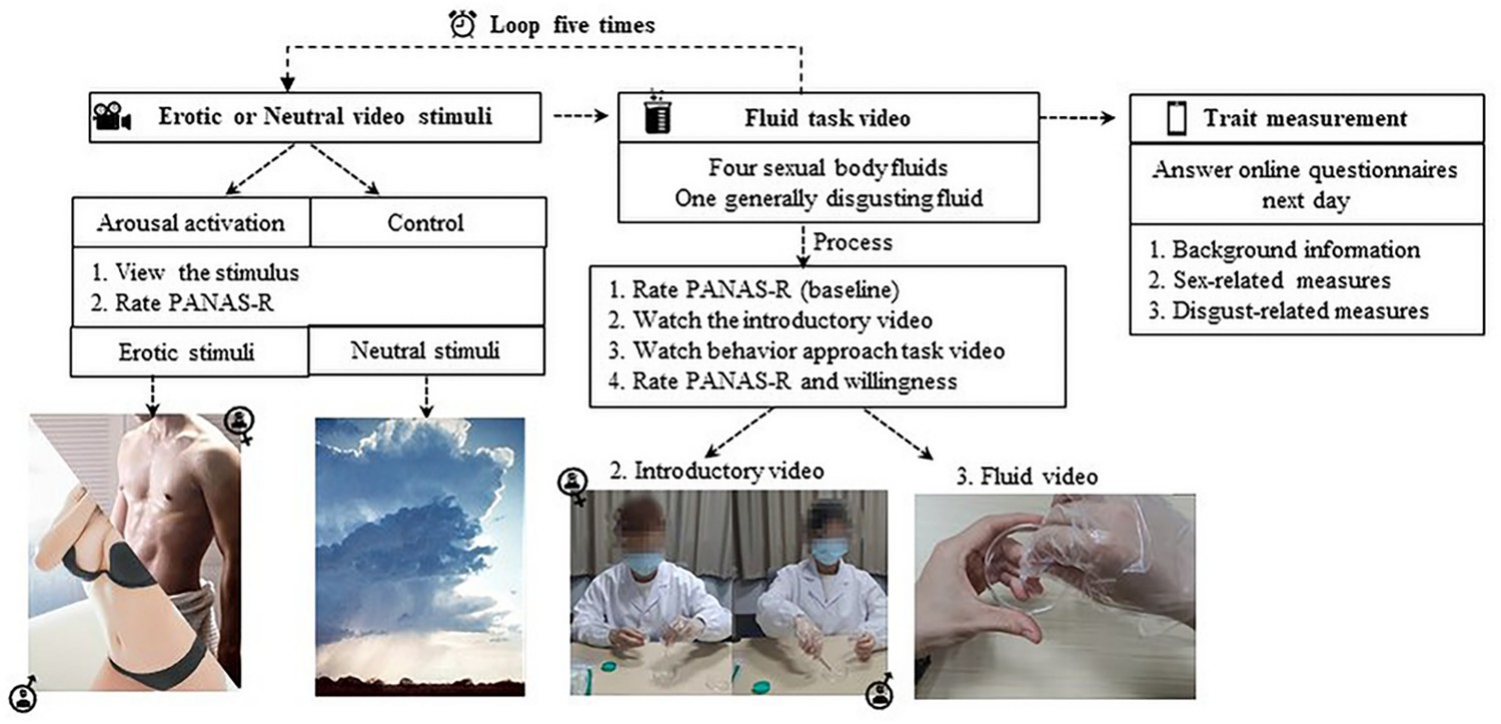
A flowchart presenting the procedure of Study 2.

Trial 1 to trial 4 presented fluid task videos regarding one of the sexual body fluids and trial 5 asked participants to view the generally disgusting fluid task. Participants finished an online survey measuring their trait characteristics via *Wenjuanxing* the next day. A flowchart of the experiment is presented in Fig 4.

*Data analysis*. Data regarding the impact of sexual body fluids and the generally disgusting material were analyzed separately. The same method as in Study 1 was used to analyze the data regarding the sexual body fluid tasks. Mixed linear models via SPSS v25.0 were used to examine whether 1) video type (erotic or neutral stimuli) affected reactions toward these stimuli, 2) video type affected reactions toward the subsequent sexual body fluid videos, 3) the effect of video type on reactions toward the sexual body fluid videos would be moderated by gender and traits characteristics.

Also, subjective ratings of sexual arousal and disgust of individuals were used via SmartPLS 3.0 to examine if 4) sexual arousal and disgust toward erotic stimuli was significantly related to disgust toward sexual body fluids as well as the willingness to finish the tasks, 5) sexual arousal state toward erotic stimuli was positively related to sexual arousal state and the willingness with sexual body fluids, 6) sexual arousal state toward sexual body fluids mediated the relationship between sexual arousal state toward erotic stimuli and the willingness to finish the sexual body fluid tasks.

We did the same analyses for data regarding the generally disgusting material using Univariate ANOVA. However, the sexual arousal state toward generally disgusting material was replaced by feelings of disgust toward generally disgusting material when we conducted the analyses using the subjective ratings because no sexual arousal was expected to be experienced when participants finished the generally disgusting material.

### Results

#### Do erotic stimuli induce feelings of sexual arousal?

Table 4 presents the effects of stimulus type (erotic video stimuli and neutral video stimuli) and gender on reactions toward these stimuli themselves as well as sexual body fluids from the mixed linear models.

**Table 4:** The effect of stimulus type on reactions to video stimuli and sexual body fluids.

**Outcome (Reactions to videos stimuli)**	**Predictor**	***df***	***F***	***p***
Sexual arousal	Stimulus type	1161.5	619.65	< .001
Disgust		1120.2	231.16	< .001
Sexual arousal state		1153.6	81.82	< .001
Sexual arousal	Gender	1161.5	83.92	< .001
Disgust		1120.2	53.88	< .001
Sexual arousal state		1153.6	135.94	< .001
Sexual arousal	Stimulus type*Gender	1161.5	58.97	< .001
Disgust		1120.2	85.82	< .001
Sexual arousal state		1153.6	138.76	< .001
**Outcome (Reactions to sexual body fluids)**	**Predictor**	***df***	***F***	***p***
Sexual arousal	Stimulus type	809.08	1.83	.176
Disgust		913.65	0.54	.465
Willingness to finish the tasks		911.79	6.71	.010
Sexual arousal state		874.73	1.35	.247
Sexual arousal	Gender	809.08	87.06	< .001
Disgust		913.65	41.66	< .001
Willingness to finish the tasks		911.79	94.73	< .001
Sexual arousal state		874.73	90.26	< .001
Sexual arousal	Stimulus type*Gender	809.08	2.13	.144
Disgust		913.65	6.34	.012
Willingness to finish the tasks		911.79	12.21	< .001
Sexual arousal state		874.73	5.93	.015

*Note*: Stimulus type refers to erotic videos or neutral videos that participants were exposed to prior to being shown sexual body fluids in subsequent tasks.

**H2.1: Exposing participants to erotic (vs. neutral) stimuli would result in higher feelings of sexual arousal, more disgust, and stronger sexual arousal state toward these stimuli. The inducing effects on sexual arousal and sexual arousal state would be more pronounced in men, whereas the disgust-inducing effect would be more pronounced in women**.

There was an effect of stimulus type on feelings of sexual arousal, disgust, and sexual arousal state (*ps* < .001). Participants viewing erotic (vs. neutral) stimuli reported higher sexual arousal (*M*_VideoErotic_ = 2.24, *SD*_VideoErotic_ = 1.21; *M*_VideoNeutral_ = 1.04, *SD*_VideoNeutral_ = 0.28), more disgust (*M*_VideoErotic_ = 1.74, *SD*_VideoErotic_ = 0.97; *M*_VideoNeutral_ = 1.11, *SD*_VideoNeutral_ = 0.44), and stronger sexual arousal state (*M*_VideoErotic_ =0.51, *SD*_VideoErotic_ = 1.69; *M*_VideoNeutral_ = -0.06, *SD*_VideoNeutral_ = 0.48).

There was a significant interaction between gender and stimulus type on sexual arousal state (*p* < .001). Men viewing erotic (vs. neutral) stimuli reported a stronger sexual arousal state (*M*_VideoErotic_ = 1.28, *SD*_VideoErotic_ =1.47; *M*_VideoNeutral_ = -0.07, *SD*_VideoNeutral_ = 0.59; *p* < .001), while there was no stimulus type effect in women (*p* = .054). There were significant interactions between gender and stimulus type on sexual arousal and disgust (*ps* < .001). However, post hoc analyses showed that both men and women who viewed erotic (vs. neutral) stimuli reported higher sexual arousal and higher disgust (*ps* < .001). Instead, men viewing erotic stimuli (vs. women viewing erotic stimuli) reported higher sexual arousal (*M*_Men_ = 2.66, *SD*_Men_ = 1.28; *M*_Women_ = 1.87, *SD*_Women_ = 0.95; *p* < .001) and less disgust (*M*_Men_ = 1.37, *SD*_Men_ = 0.69; *M*_Women_ = 2.07, *SD*_Women_ = 1.06; *p* < .001), while there was no significant gender difference on feeling of sexual arousal and disgust (*ps* > .071) in participants viewing neutral stimuli.

**H2.1b: Men (vs. women) would report higher sexual arousal, less disgust, and stronger sexual arousal state toward the stimuli**.

Gender had main effects on feelings of sexual arousal, disgust, and sexual arousal state (*ps* <.001). Men reported higher sexual arousal (*M*_Men_ = 1.84, *SD*_Men_ = 1.24; *M*_Women_ = 1.44, *SD*_Women_ = 0.80), less disgust (*M*_Men_ = 1.26, *SD*_Men_ = 0.62; *M*_Women_ = 1.58, *SD*_Women_ = 0.95), and had stronger sexual arousal state after viewing the stimuli (*M*_Men_ = 0.58, *SD*_Men_ = 1.30; *M*_Women_ = -0.15, *SD*_Women_ = 1.13) compared to women.

### The effect of erotic stimuli on reactions toward sexual body fluid tasks

#### Do erotic stimuli affect reactions to sexual body fluid tasks?

Table 4 also presents the effects of stimulus type and gender on reactions toward the subsequent sexual body fluid tasks.

**H2.2: Disgust elicited by subsequent sexual body fluid tasks would be reduced, whereas sexual arousal, sexual arousal state, and willingness to finish the tasks elicited by sexual body fluid tasks would be increased after prior exposure to erotic (vs. neutral) stimuli. The effects of erotic stimuli on feelings toward subsequent sexual body fluid tasks would be more pronounced in men compared to women**.

Stimulus type had a main effect on the willingness to finish subsequent sexual body fluid tasks (*p* = .01), with participants viewing erotic (vs. neutral) stimuli reporting higher willingness to finish them (*M*_VideoErotic_ = 3.84, *SD*_VideoErotic_ = 2.41; *M*_VideoNeutral_ = 3.52, *SD*_VideoNeutral_ = 2.27). Video type did not affect feelings of sexual arousal (*p* = .176; *M*_VideoErotic_ = 1.37, *SD*_VideoErotic_ = 0.84; *M*_VideoNeutral_ = 1.31, *SD*_VideoNeutral_ = 0.74), disgust (*p* = .465; *M*_VideoErotic_ = 2.31, *SD*_VideoErotic_ = 1.24; *M*_VideoNeutral_ = 2.36, *SD*_VideoNeutral_ = 1.21) or sexual arousal state toward the sexual body fluid tasks (*p* = .247; *M*_VideoErotic_ = -0.94, *SD*_VideoErotic_ = 1.59; *M*_VideoNeutral_= -1.05, *SD*_VideoNeutral_ = 1.47) even though the means were in the expected direction.

**Fig 5:**
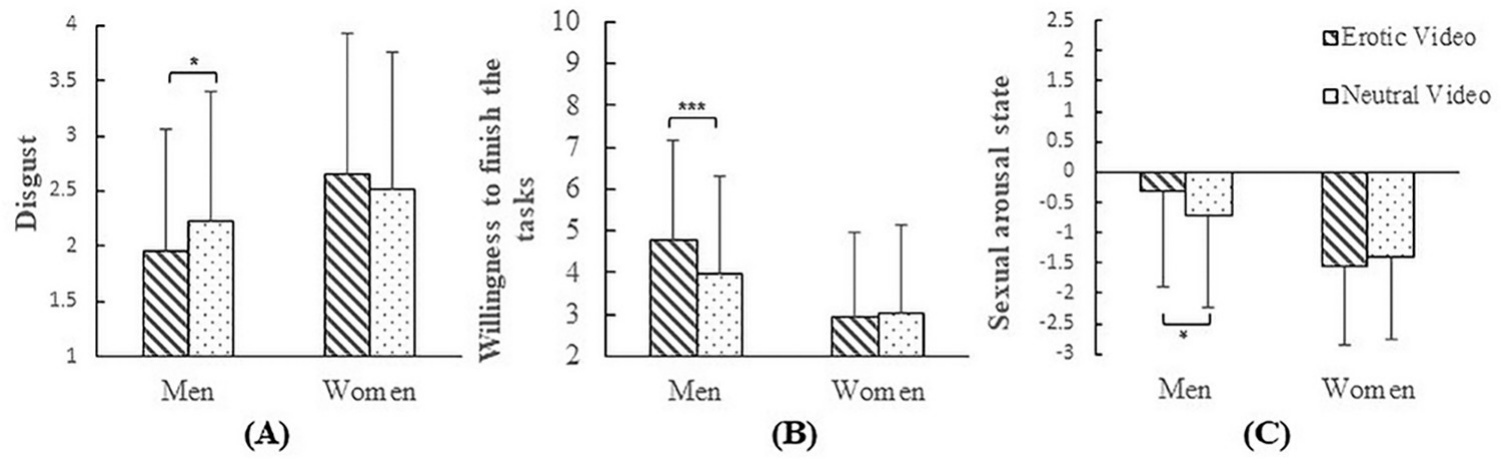
The interaction between stimulus type and gender on reactions to sexual body fluid tasks. *Note*: * *p* < .05, *** *p* < .001.

There were significant interactions between gender and stimulus type on feelings of disgust (*p* = .012; See Fig 5A), the willingness to finish these sexual body fluid tasks (*p* < .001; See Fig 5B), and sexual arousal state (*p* = .015; See Fig 5C), with men viewing erotic (vs. men viewing neutral) stimuli reporting less disgust (*M*_VideoErotic_ = 1.95, *SD*_VideoErotic_ = 1.11; *M*_VideoNeutral_ = 2.22, *SD*_VideoNeutral_ = 1.18; *p* = .022), higher willingness to finish these sexual body fluid tasks (*M*_VideoErotic_ = 4.80, *SD*_VideoErotic_ = 2.38; *M*_VideoNeutral_ = 3.96, *SD*_VideoNeutral_ = 2.34; *p* < .001) as well as stronger sexual arousal state (*M*_VideoErotic_ = -0.30, *SD*_VideoErotic_ = 1.60; *M*_VideoNeutral_ = -0.72, *SD*_VideoNeutral_ = 1.52; *p* = .011), while there was no effect of stimulus type in women on disgust, willingness to finish these tasks, and sexual arousal state (*ps* > .208). There was no interaction between gender and stimulus type on sexual arousal toward sexual body fluid tasks (*p* = .144).

**H2.2b: Women would report more disgust, lower sexual arousal, weaker sexual arousal state, lower willingness to finish the tasks toward the sexual body fluid tasks compared to men**.

Gender had main effects on feelings of sexual arousal, disgust, sexual arousal state, and willingness to finish the sexual body fluid tasks (*ps* <.001). Men reported higher sexual arousal (*M*_Men_ = 1.57, *SD*_Men_ = 1.00; *M*_Women_ = 1.11, *SD*_Women_ = 0.38), less disgust (*M*_Men_ = 2.09, *SD*_Men_ = 1.15; *M*_Women_ = 2.59, *SD*_Women_ = 1.25), stronger sexual arousal state (*M*_Men_ = -0.52, *SD*_Men_ = 1.57; *M*_Women_ = -1.48, *SD*_Women_ = 1.33), and a higher willingness to finish the sexual body fluid tasks (*M*_Men_ = 4.37, *SD*_Men_ = 2.39; *M*_Women_ = 2.98, *SD*_Women_ = 2.07).

#### Do sexuality and disgust-related trait characteristics moderate the effects of sexual arousal induced by erotic stimuli on reactions to sexual body fluid tasks?

Table 5 presents the results of interactions between stimulus type, trait characteristics, and gender on reactions to sexual body fluid tasks.

**Table 5:** The interaction between stimulus type, trait characteristics, and gender on reactions to sexual body fluid tasks.

Outcome^a^	Predictor	*df*	*F*	*p*
Sexual arousal	Stimulus type*Sexual excitability	750.22	7.38	.007
Disgust		836.26	0.87	.352
Willingness to finish the tasks		839.54	0.07	.799
Sexual arousal state		812.95	3.91	.048
Sexual arousal	Stimulus type*Sexual excitability*Gender	750.22	4.61	.032
Disgust		836.26	1.44	.230
Willingness to finish the tasks		839.54	0.41	.524
Sexual arousal state		812.95	4.30	.038
Sexual arousal	Stimulus type*Sexual disgust sensitivity	750.31	1.01	.316
Disgust		835.23	2.02	.156
Willingness to finish the tasks		839.50	2.53	.112
Sexual arousal state		807.44	0.57	.452
Sexual arousal	Stimulus type*Sexual disgust sensitivity*Gender	750.31	0.73	.394
Disgust		835.23	0.48	.488
Willingness to finish the tasks		839.50	0.70	.404
Sexual arousal state		807.44	1.35	.245

*Note*: ^a^ = Reactions to sexual body fluid tasks. Stimulus type refers to erotic videos or neutral videos that participants were exposed to prior to being shown sexual body fluids in subsequent tasks.

**H2.3 The effects of erotic stimuli on feelings toward subsequent sexual body fluid tasks would be more pronounced in participants with more (vs. less) sexual excitability and those with less (vs. more) sexual disgust sensitivity**

Regarding the moderating effect of sexual excitability, there were significant interactions between video type and sexual excitability as well as the interaction between stimulus type, gender, and sexual excitability on sexual arousal (*p* = .007; *p* = .032) and sexual arousal state (*p* = .048; *p* = .038) toward sexual body fluid tasks. Men with high sexual excitability viewing erotic (vs. neutral) stimuli reported higher feelings of sexual arousal (*M*_VideoErotic_ = 1.89, *SD*_VideoErotic_ = 1.21; *M*_VideoNeutral_ = 1.55, *SD*_VideoNeutral_ = 0.94; *p* < .001) and higher sexual arousal state toward sexual body fluid tasks (*M*_VideoErotic_ = 0.13, *SD*_VideoErotic_ = 1.67; *M*_VideoNeutral_ = -0.62, *SD*_VideoNeutral_ = 1.40; *p* < .001), while there was no stimulus type difference in men with low sexual excitability or women with low or high sexual excitability (*ps* > .106). There was no interaction between stimulus type and sexual excitability (*ps* > . 352) nor an interaction between stimulus type, sexual excitability, and gender (*ps* > . 524) on disgust or willingness to finish the sexual body fluid tasks.

Regarding the moderating effect of sexual disgust sensitivity, there was no significant interaction between stimulus type and sexual disgust sensitivity (*ps* > .112) or interaction between stimulus type, sexual disgust sensitivity, and gender (*ps* > .245) on feelings of sexual arousal, disgust, sexual arousal state, or the willingness to finish sexual body fluid tasks.

#### Are the subjective feelings of sexual arousal and disgust induced by erotic stimuli associated with disgust and the willingness to finish sexual body fluid tasks?

**Fig 6:**
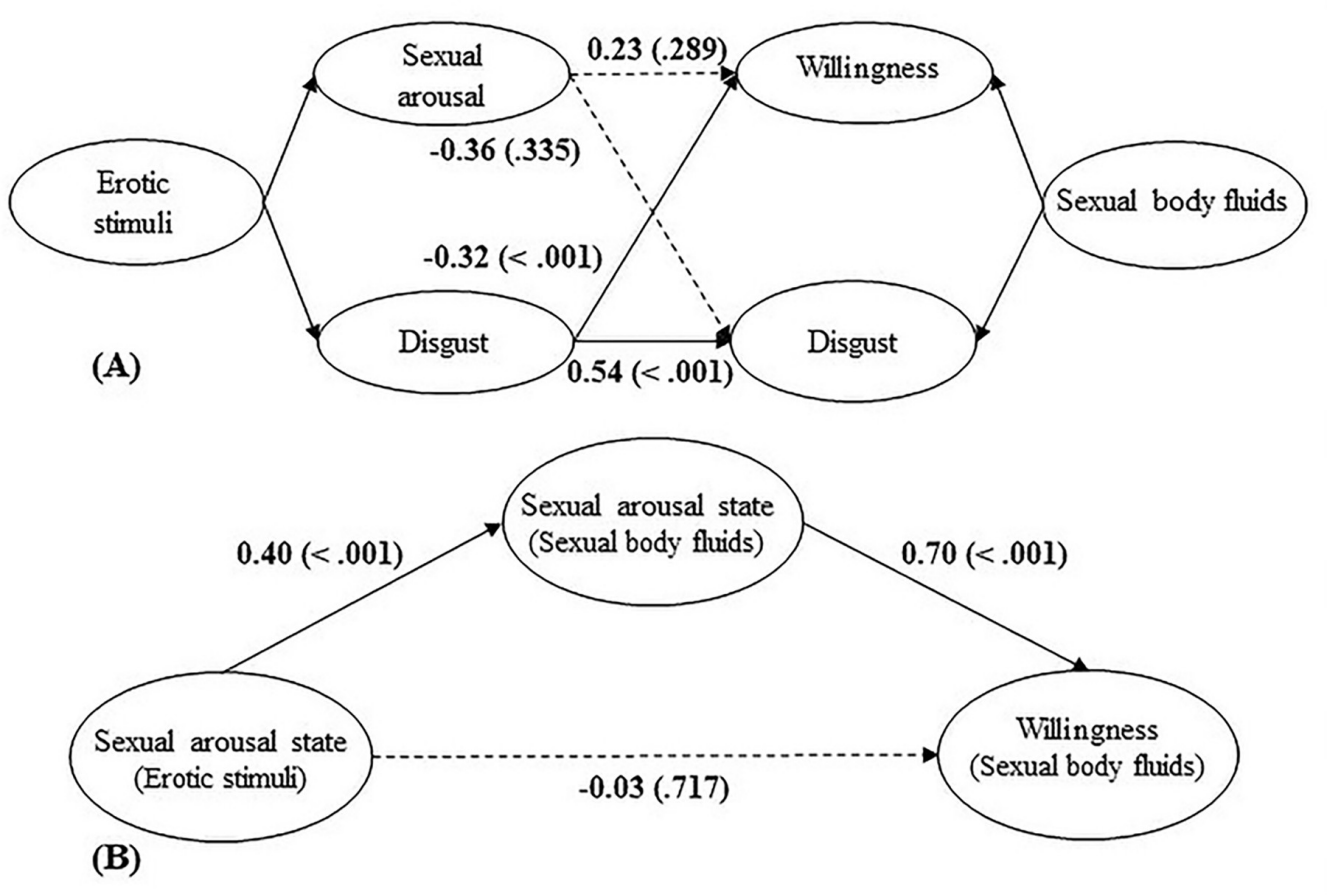
The associations between reactions to erotic stimuli and reactions to sexual body fluids. *Note*: Number outside the brackets = *β*; Number in the brackets = *p*-value.

Fig 6 presents the associations between subjective feelings toward erotic stimuli and reactions to sexual body fluids.

**H 2.4.1 Sexual arousal toward erotic stimuli would be negatively related to disgust toward the sexual body fluids and the willingness to finish the tasks**.

As presented in Fig 6A, sexual arousal toward erotic stimuli was not related to disgust toward sexual body fluids and the willingness to finish the tasks (*ps* > .289).

**H 2.4.2 Disgust toward erotic stimuli would be positively related to feelings of disgust toward sexual body fluids but negatively related to the willingness to finish these tasks**.

As expected, results showed that disgust toward erotic stimuli was positively related to disgust toward sexual body fluids and negatively related to the willingness to finish these tasks (*ps* < .001).

#### Does sexual arousal state toward erotic stimuli affect sexual arousal state and the willingness to finish sexual body fluid tasks?

**H 2.5.1 Sexual arousal state toward the erotic stimuli would be positively related to sexual arousal state toward the sexual body fluid tasks and the willingness to finish these tasks**.

Regression analyses showed that sexual arousal state toward erotic stimuli was positively related to sexual arousal state toward sexual body fluid tasks (*β* = 0.40, *p* < .001) and the willingness to finish these tasks (*β* = 0.25, *p* = .014).

**H 2.5.2 Sexual arousal state toward sexual body fluids tasks would mediate the relationship between sexual arousal state toward erotic stimuli and the willingness to finish the sexual body fluid tasks**.

As presented in Fig 6B, sexual arousal state toward erotic stimuli was positively related to sexual arousal state toward the sexual body fluid tasks (*p* < .001), while there was a positive relationship between sexual arousal state toward sexual body fluid tasks and the willingness to finish these tasks (*p* < .001). Sexual arousal state toward sexual body fluids tasks mediated the relationship between sexual arousal state toward erotic stimuli and the willingness to finish the sexual body fluid tasks and the standardized indirect effect was 0.28 (95%CI [.15, .89]).

### The effect of erotic stimuli on reactions toward generally disgusting task

#### Do erotic stimuli affect reactions toward generally disgusting fluid task?

Table 6 presents the effects of stimulus type and gender on reactions toward the generally disgusting task.

**Table 6:** The effect of video type and gender on reactions to the generally disgusting fluid task.

Outcome^a^	Predictor	*df*	*F*	*p*
Sexual arousal	Stimulus type	231	0.45	.505
Disgust		231	1.85	.175
Willingness to finish this task		231	0.00	.967
Sexual arousal state		231	1.12	.292
Sexual arousal	Gender	231	3.32	.070
Disgust		231	2.43	.120
Willingness to finish this task		231	0.68	.411
Sexual arousal state		231	0.78	.378
Sexual arousal	Stimulus type*Gender	231	1.02	.313
Disgust		231	2.82	.094
Willingness to finish this task		231	0.43	.515
Sexual arousal state		231	3.53	.062

Note

^a^ = Reactions to generally disgusting fluid task. Stimulus type refers to erotic videos or neutral videos that participants were exposed to prior to being shown generally disgusting fluid in subsequent task.

**H2.2: Disgust elicited by the generally disgusting fluid task would be reduced, whereas willingness to finish the generally disgusting fluid task would be increased after prior exposure to erotic (vs. neutral) stimuli. The effects of erotic stimuli on reactions toward the generally disgusting task would be more pronounced in men compared to women**.

Stimulus type did not affect feelings of sexual arousal, disgust, sexual arousal state, or the willingness to finish the generally disgusting fluid task (*ps* > .175).

There was no interaction between gender and stimulus type on feelings of sexual arousal, disgust, sexual arousal state toward the generally disgusting fluid task, or the willingness to finish this task (*ps* > .062).

**H2.2b: Women would report more disgust and lower willingness to finish the task toward the generally disgusting task compared to men**.

Gender did not affect the feelings of sexual arousal, disgust, sexual arousal state, or the willingness to finish the generally disgusting fluid task (*ps* > .070).

#### Do sexuality and disgust-related trait characteristics affect the effect of erotic stimuli on reactions to the generally disgusting fluid task?

Table 7 presents the results of interactions between stimulus type, trait characteristics, and gender on reactions to the generally disgusting fluid task.

**Table 7:** The interaction between stimulus type, trait characteristics, and gender on reactions to the generally disgusting fluid task.

Outcome^a^	Predictor	*df*	*F*	*p*
Sexual arousal	Stimulus type*Sexual excitability	209	0.07	.787
Disgust		209	2.07	.152
Willingness to finish this task		209	2.44	.120
Sexual arousal state		209	1.63	.204
Sexual arousal	Stimulus type*Sexual excitability*Gender	209	0.63	.429
Disgust		209	1.02	.314
Willingness to finish this task		209	0.61	.434
Sexual arousal state		209	1.40	.238
Sexual arousal	Stimulus type*Sexual disgust sensitivity	209	0.01	.935
Disgust		209	1.03	.311
Willingness to finish this task		209	0.76	.384
Sexual arousal state		209	0.96	.328
Sexual arousal	Stimulus type*Sexual disgust sensitivity*Gender	209	0.03	.858
Disgust		209	0.88	.350
Willingness to finish this task		209	0.00	.971
Sexual arousal state		209	0.69	.408

Note

^a^ = Reactions to generally disgusting fluid task. Stimulus type refers to erotic videos or neutral videos that participants were exposed to prior to being shown generally disgusting fluid in subsequent task.

**H2.3 The effects of erotic stimuli on feelings toward subsequent generally disgusting fluid task would be more pronounced in participants with more (vs. less) sexual excitability and those with less (vs. more) sexual disgust sensitivity**.

Regarding the moderating effect of sexual excitability, there was no interaction between stimulus type and sexual excitability (*ps* > .120) nor any interaction between stimulus type, sexual excitability, and gender (*ps* > .238) on sexual arousal, disgust, sexual arousal state toward the generally disgusting fluid task, or the willingness to finish this task.

Regarding the moderating effect of sexual disgust sensitivity, there was no interaction between stimulus type and sexual disgust sensitivity (*ps* > .311) nor interactions between stimulus type, sexual disgust sensitivity, and gender (*ps* > .350) on feelings of sexual arousal, disgust, sexual arousal state, or willingness to finish the generally disgusting fluid task.

#### Are the subjective feelings of sexual arousal and disgust induced by erotic stimuli associated with disgust and the willingness to finish the generally disgusting fluid task?

**Fig 7:**
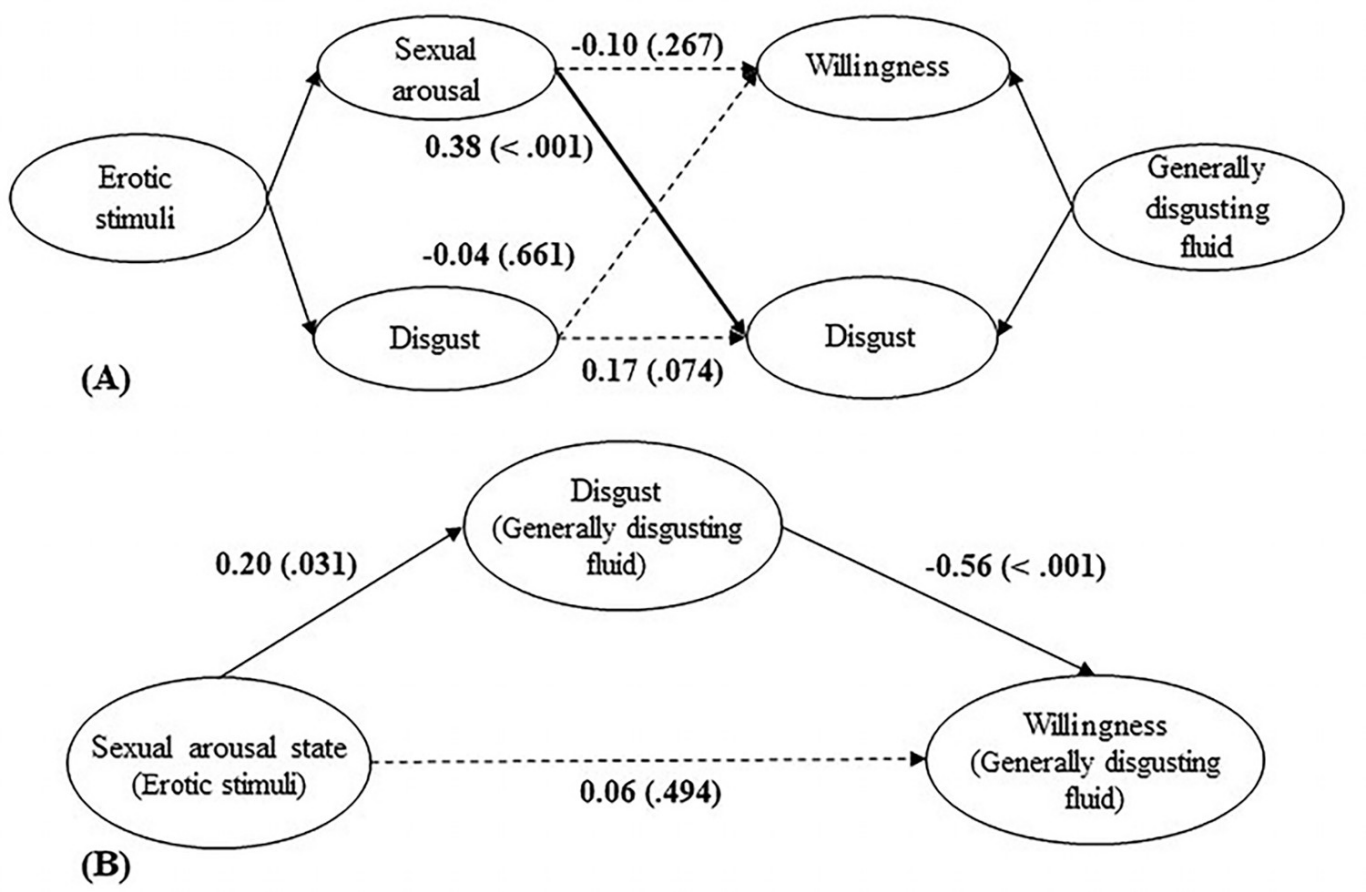
The associations between reactions to erotic stimuli and reactions to the generally disgusting fluid task. *Note*: Number outside the brackets = *β*; Number in the brackets = *p*-value.

Fig 7 presents the associations between subjective feelings toward erotic stimuli and reactions to the generally disgusting fluid task.

**H 2.4.1 Sexual arousal toward erotic stimuli would be negatively related to disgust but positively related to the willingness toward the generally disgusting fluid task**.

As presented in Fig 7A, sexual arousal toward erotic stimuli was positively related to disgust toward the generally disgusting fluid task (*p* < .001) but not related to the willingness to finish the task (*p* = .267).

**H 2.4.2 Disgust toward erotic stimuli would be positively related to feelings of disgust toward the generally disgusting fluid task but negatively related to the willingness to finish this task**.

Results showed that disgust toward erotic stimuli was neither related to disgust (*p* = .074) nor willingness to finish this generally disgusting fluid task (*p =* .661).

#### Does sexual arousal state toward erotic stimuli affect disgust and the willingness toward the generally disgusting fluid task?

First, a single *t*-test was used to explore whether the generally disgusting fluid task induced feelings of sexual arousal. Given that the experimental manipulation (viewing erotic stimuli) may have affected participants’ feelings toward subsequent stimuli, only data from the control group were used. The results showed that participants in the control group (who viewed the neutral stimuli) did not have a score (*M* = 1.076; *SD* = 0.417) higher than 1 (the minimum possible score on the PANAS-R, which ranged from 1 to 5) given that 95% CI of the effect size include zero (*t*(117) = 1.988, *p* = .049, *d* = 0.18, 95% CI [0, .36]). Thus, the measure of sexual arousal state was replaced by the measure of disgust induced by the generally disgusting fluid task.

**H 2.5.1 Sexual arousal state toward erotic stimuli would be negatively related to disgust toward the generally disgusting fluid task and positively related to the willingness to finish this task**.

Regression analyses showed that sexual arousal state toward erotic stimuli was positively related to disgust toward the generally disgusting task (*β* = 0.20, *p* = .031) but not related to the willingness to finish this task (*β* = -0.06, *p* = .544).

**H 2.5.2 Disgust toward the generally disgusting fluid task would mediate the relationship between sexual arousal state toward erotic stimuli and the willingness to finish the generally disgusting fluid task**.

As presented in Fig 7B, sexual arousal state toward erotic stimuli was positively related to disgust toward the generally disgusting fluid task (*p* = .031), while feelings of disgust and the willingness toward the generally disgusting task were negatively related (*p* < .001). Disgust toward the generally disgusting fluid task mediated the relationship between sexual arousal state toward erotic stimuli and the willingness toward the generally disgusting fluid task and the standardized indirect effect was -0.11 (95%CI [-.34, -.01]).

## Discussion

The current research explored the bidirectional relationship between sex-related disgust induced by sexual body fluids and sexual arousal toward erotic stimuli. We were also interested in whether gender and relevant trait characteristics moderated these effects of manipulation.

Specifically, Study 1 presented the participants with sexual body (vs. neutral) fluids followed by erotic stimuli. The primary findings showed that the presentation of sexual body fluids inhibited sexual arousal and the likelihood of sexual engagement toward erotic stimuli in participants with high sexual disgust sensitivity but increased sexual arousal and the likelihood of sexual engagement in participants with low sexual disgust sensitivity. Study 2 presented the participants with erotic (vs. neutral) videos followed by sexual body fluids (and a non-sex-related stimulus). Results showed that men exposed to erotic stimuli reported lower disgust, stronger sexual arousal state, and higher willingness to interact with the sexual body fluids.

We did not find a significant negative relationship between subjective feelings of sexual arousal and disgust in these two experiments, but the balance of sexual arousal and disgust (i.e., sexual arousal state) toward sexual body fluids and the erotic stimuli had a positive association.

Also, erotic stimuli failed to affect reactions toward the generally disgusting fluid task, while, in an unexpected finding, subjective feelings of sexual arousal and sexual arousal state toward erotic stimuli were positively related to feelings of disgust toward the generally disgusting fluid task.

### Feelings induced by sexual body fluids (Study 1) and erotic stimuli (Study 2)

As expected (H1.1), we found that sexual body fluid tasks successfully induced higher disgust, weaker sexual arousal state, and a lower willingness to finish the tasks in Study 1. This is consistent with previous evolutionary hypotheses suggesting that body fluids, like sperm and vaginal secretions, are associated with contagion risk and therefore elicit feelings of disgust and possibly sexual avoidance behavior because of the activation of the Behavioral Immune System [10, 15]. Also, erotic stimuli elicited higher sexual arousal and higher sexual arousal state in Study 2, also in line with our expectations (H 2.1). This indicates that our manipulations were successful and videos presenting sexual body fluids could be used to activate disgust. Interestingly, the presentation of sexual body fluid tasks also induced feelings of sexual arousal in men, while erotic stimuli elicited feelings of disgust in both men and women. This supports the model developed by de Jong et al. (2013) suggesting that sexual stimuli induce both sexual arousal and disgust.

### Effects of sexual body fluids on reactions toward subsequent erotic stimuli

Contrary to what we expected (H1.2), exposure to sexual body fluids did not affect feelings of sexual arousal, disgust, sexual arousal state toward erotic stimuli, or the likelihood of sexual engagement toward these stimuli.

From an evolutionary perspective, sexual arousal and disgust are seen to be contradictory, with disgust expected to suppress sexual arousal and elicit sexual avoidance behavior to protect individuals from pathogen risk, and sexual arousal expected to inhibit experiences of disgust in order for mating and reproduction to occur [10]. Previous experimental evidence has shown that upregulation of disgust (e.g., exposure to disgusting pictures or odors) reduced feelings of sexual arousal in men and women [16, 21]. However, the current study failed to discover the suppressing effect of disgust on subsequent sexual arousal and sexual approach behavior. One possibility is that the types of stimuli used did not elicit strong enough disgust. Although the stimuli depicted the process of smelling and touching sexual body fluids and therefore be considered to have higher ecological validity and mimic actual sexual encounters better than previous manipulations, these stimuli were only presented visually, which may have limited their realness. Also, previous sexual experience may be an essential factor in affecting the impact of body fluids. Compared to those with few sexual experiences, participants with rich experiences would feel less disgusted by these stimuli because they have been exposed to these stimuli more frequently. Thus, future experiments should consider using direct contact with sexual body fluids and sexual experience-related individual differences in the analyses.

Another possibility is that the effects of disgust on reactions toward erotic stimuli are moderated by trait characteristics. Consistent with what we expected (H1.3), we found that participants with high disgust sensitivity reported lower sexual arousal and lower likelihood of sexual engagement toward the erotic stimuli after viewing the sexual body (vs. neutral) fluid videos. This is in line with the theoretical framework proposed by de Jong et al. (2013) suggesting that trait-related disgust moderates the strength of disgust elicited by sexual stimuli [10]. Thus, when participants with high disgust sensitivity encounter sexual body fluids, their experience of disgust may be strong enough to inhibit their sexual arousal and sexual approach behavior.

However, we also found that participants with low disgust sensitivity reported higher sexual arousal and higher likelihood of sexual engagement toward the erotic stimuli after viewing the sexual body (vs. neutral) fluids. This suggests that these participants experienced both sexual arousal and disgust from sexual body fluids, and the increase in sexual arousal toward sexual body fluids then increased their sexual arousal toward erotic stimuli possibly through priming and/or accumulative effects.

### Effects of erotic stimuli on reactions to sexual body fluids

We found that participants overall reported higher willingness to finish the sexual body fluid tasks after viewing erotic stimuli, which is in line with our expectations (H2.2), while there was no effect on feelings of disgust, even though the feelings of disgust toward sexual body fluids after viewing erotic (vs. neutral) stimuli showed a decreasing tendency. Also, it was men who reported lower disgust, higher willingness to finish sexual body fluid tasks, and a stronger sexual arousal state toward sexual body fluids after viewing erotic (vs. neutral) stimuli, while there were no effects of erotic stimuli in women.

Gender differences in experiencing feelings of sexual arousal toward erotic stimuli might contribute to this gender difference. Parental investment theory suggests that compared to men, women have greater mating and rearing investment so that they develop higher disgust and higher sexual inhibition tendencies, referring to being choosy when they select sexual partners, while men would be more promiscuous to have more sexual partners to benefit offspring [36]. Current studies also found that men reported higher sexual arousal and sexual arousal state toward both sexual body fluid tasks and erotic stimuli, while women reported higher disgust toward these two types of stimuli. Thus, compared to women, men experiencing higher sexual arousal would be more likely to experience low disgust toward sex-related disgusting stimuli. Another possible reason is that the strength of sexual arousal elicited by sexual stimuli in Study 2 (static images) is not strong enough to work in women given the lower sexual excitability of women, while Borg et al.(2012) used erotic films to elicit higher sexual arousal [16].

We expected relevant trait characteristics to affect how erotic stimuli impact reactions to sexual body fluids. However, different from our expectation (H2.3), results showed that men with higher sexual excitability reported higher sexual arousal and higher sexual arousal state toward sexual body fluids after viewing the erotic stimuli. A likely reason is that men with high sexual excitability experienced the highest sexual arousal, and this sexual arousal state extended across stimuli and elicited sexual arousal toward sexual body fluids, given that there was no increase in sexual arousal toward sexual body fluids in men with high sexual excitability viewing neutral videos or men with low sexual excitability viewing erotic stimuli. This result can be understood in light of the sexual arousal-disgust rival model, wherein sexual excitability moderates the intensity of sexual arousal from sexual stimuli and affects the suppressing effects of sexual arousal on disgust.

### The specificity of the moderating effects of traits during the interplay between sexual arousal and disgust

The sexual arousal-disgust rival model suggests that disgust propensity moderates the intensity of disgust elicited by sexual stimuli, while sexual excitability moderates the intensity of sexual arousal [10]. In these experiments, we had the opportunity to see whether disgust-related traits moderate the experience of sexual arousal and whether sexual-related traits moderate the experience of disgust from sexual stimuli. Previous evidence suggests that disgust sensitivity moderates the feelings of sexual arousal toward erotic stimuli while evidence on possible moderating effects of sexual excitability on disgust is lacking [40, 41]. Our results showed that sexual excitability had no moderating effects on the effects of disgust induced by sexual body fluids on sexual arousal, while disgust sensitivity could not moderate the suppressing effects of sexual arousal elicited by erotic stimuli on disgust induced by sexual body fluids. This suggests that the moderating effects of these two traits are likely to be specific. These and the above findings strengthen the conclusion that trait characteristics and gender play essential roles in the relationship between sexual arousal and disgust.

### Bidirectional relationship between subjective feelings of sexual arousal toward erotic stimuli and disgust induced by sexual body fluids

The interplay between sexual arousal and sex-related disgust is a complex process given that both sexual stimuli and sexual body fluids induce both sexual arousal and disgust. Thus, it is important to explore the relationship between subjective feelings of sexual arousal and disgust besides the analyses exploring the effects of experimental manipulations that induce both sexual arousal and disgust, which can cause counterintuitive results. We hypothesized that sexual arousal elicited by erotic stimuli would inhibit disgust toward disgusting stimuli, whereas disgust elicited by erotic stimuli would increase disgust toward disgusting stimuli. Also, disgust induced by sexually disgusting stimuli would suppress feelings of sexual arousal toward erotic stimuli, while sexual arousal elicited by sexually disgusting stimuli would enhance subsequent feelings of sexual arousal.

Different from our expectations (H1.4 and H2.4), we found that sexual arousal rather than disgust toward sexual body fluids showed a significant positive relationship to sexual arousal and the likelihood of sexual engagement toward subsequent erotic stimuli, and disgust rather than sexual arousal toward erotic stimuli showed a significant relationship with experiences of disgust and willingness to finish the sexual body fluid tasks.

Although the current studies did not find a significant negative relationship between sexual arousal and disgust, the findings inform us of the mechanisms behind this interplay and why some research failed to verify these negative relationships, with sexual arousal elicited by disgusting stimuli interrupting the effects of disgust on reactions toward erotic stimuli and disgust elicited by erotic stimuli interrupting effects of sexual arousal on reactions to sexual body fluids as priming and/or accumulative effects.

Furthermore, these findings regarding subjective feelings point to the dangers of exploring only one outcome emotion, without taking into account the other emotion elicited by the same stimuli, such as sexual arousal elicited by sexual body fluids, also when behavioral outcomes are looked at [10]. The current studies also calculated the balance between sexual arousal and disgust toward the same stimuli to indicate the sexual arousal state after receiving the manipulations and explored how this arousal state affected their reactions to the subsequent stimuli. The findings showed a positive relationship between sexual arousal state toward sexual body fluids and sexual arousal state toward erotic stimuli, and the sexual arousal state toward the manipulation affected the behavioral intentions toward the latter stimuli via increasing their sexual arousal state toward these latter stimuli.

### Effects of erotic stimuli on reactions to the generally disgusting fluid task

Previous evidence regarding the effects of sexual arousal on non-sex-related disgust is conflicting. Stevenson et al. (2011) failed to find a suppressing effect of sexual arousal on disgust in male undergraduates [27], while exposure to erotic stimuli could inhibit feelings of disgust and willingness to finish non-sex-related tasks in women [16]. The current studies found that neither men nor women showed decreases in disgust toward the generally disgusting fluid or increases in willingness to finish this task after the sexual arousal manipulation. This indicates that the suppressing effects of erotic stimuli would seem to be specific to sexual activity. Such a carefully evolved mechanism would be beneficial because it limits the effects of sexual arousal on sexuality-related stimuli to restrict unnecessary attraction and arousal to targets that contain the risk of pathogens and allows disgust to other contagion sources to work appropriately when sexual arousal occurs to protect people from non-sexual-related contagion.

Interestingly, we found that sexual arousal toward erotic stimuli was positively related to disgust toward the generally disgusting fluid. This was in contrast to our expectations (H2.2). One possible explanation would be excitation transfer. Excitation transfer refers to a situation where a lingering emotional response is combined with the excitatory reaction to a new stimulus, intensifying the emotional response [49]. In Study 2, erotic stimuli may have caused both sexual and general arousal of the body and this latter activation may have enhanced feelings of disgust toward subsequent stimuli. Participants may have misunderstood these body activations elicited by erotic stimuli as an experience of disgust toward the generally disgusting fluid. Similarly, Schippers et al. (2022) found that male participants rated disgusting films as emotionally arousing [49]. Though the mechanism regarding the enhancing effects of sexual arousal on non-sex-related disgust is unclear, it is meaningful for us to understand the occurrence of unusual sexual interest. Further research is needed to explore why sexual arousal works in a different direction for sex-related disgust and non-sex-related disgust.

### Strength and limitation

The current research is the first to use sexual body fluids as actual contamination-related stimuli associated with sexual activity to explore the bidirectional interplay between sexual arousal and disgust. We also measured trait characteristics and investigated how gender and traits influenced this interaction. Measures of subjective feelings of both sexual arousal and disgust toward the manipulation and subsequent stimuli, as well as the new variable named "sexual arousal state," allow us to detect the relationship between sexual arousal and disgust and its effects on behavioral outcomes further.

This research has some limitations. First, the use of heterosexual and young college samples with considerable participants who experienced little sexual activity limits the generalization to real-life situations. Research should have better controls or explore the roles of this background information (e.g., age, sexual orientation, sexual experience, and general physical health) that may affect the interplay between sexual arousal and disgust. Second, the stimuli used to induce sex-related disgust and non-sex-related disgust are only presented visually, which may not induce strong enough emotions and cause a failure to detect the effects of manipulations. Future studies should consider using direct contact with sexual body fluids. Also, the erotic stimuli used in current studies are static pictures of nude and semi-nude models from the preferred sex because of the restrictions on the use of porn, which may reduce the effectiveness of the manipulations. Research should use the same and more effective erotic stimuli that induce stronger sexual arousal, like porn clips, for men and women. Finally, a repeated measure design was used in the current research, and participants needed to finish the tasks four to five times one by one, which means that the feelings they experienced from the latter stimuli in the prior trial would interrupt the effectiveness of the manipulation in the next trial. Future studies could use a between-subject design or a better methodology (e.g., cross-lagged panel models) to detect only the relationship between these two target emotions.

## Conclusion

The current research employed two experiments to explore the bidirectional relationship between sexual arousal toward erotic stimuli and disgust induced by sexual body fluids. The findings showed that exposure to sex-related disgust stimuli suppressed feelings of sexual arousal and the likelihood of sexual engagement toward subsequent erotic stimuli in participants with high sexual disgust sensitivity but increased these reactions in participants with low sexual disgust sensitivity. Study 2 found that men reported lower feelings of disgust, stronger sexual arousal state toward sexual body fluids, and higher willingness to finish these tasks after viewing erotic (vs. neutral) stimuli. Correlation analyses showed that the subjective feelings of sexual arousal but not disgust toward sexual body fluids were positively related to sexual arousal and sexual engagement toward erotic stimuli, while the feelings of disgust rather than sexual arousal toward erotic stimuli were associated with disgust and willingness to finish the sexual body fluid tasks. We also found that sexual arousal state toward the manipulation showed a positive relation to sexual arousal state toward subsequent stimuli. Finally, exposure to erotic stimuli showed no effects on reactions toward generally disgusting fluid, but there was a positive relationship between sexual arousal toward erotic stimuli and disgust toward generally disgusting fluid.

## Supporting information

S1 Data Data of Study 1 and Study 2. (ZIP)
